# Supplementary material for: 3D-QSAR, Molecular Docking and Molecular Dynamics Simulation of Pseudomonas aeruginosa LpxC Inhibitors
Source: Int J Mol Sci. 2017 May 6;18(5):761. doi: 10.3390/ijms18050761 (PMC5454807; doi:10.3390/ijms18050761)
Supplement: Supplementary file 1 [file ijms-18-00761-s001.zip › ijms-180988-final supp.docx]

Supplementary Materials: 3D-QSAR, Molecular Docking and Molecular Dynamics Simulation of *Pseudomonas* *aeruginosa* LpxC Inhibitors

Ke Zuo, Li Liang, Wenyi Du, Xin Sun, Wei Liu, Xiaojun Gou, Hua Wan and Jianping Hu

**Table S1.** Statistical parameters of the comparative molecular filed analysis (CoMFA) and comparative molecular similarity index analysis (CoMSIA) models.

| **Models** | **Partial Least Square (PLS) Statistics** | | | | | | | | | **Contributions** | | | | |
| --- | --- | --- | --- | --- | --- | --- | --- | --- | --- | --- | --- | --- | --- | --- |
|  | *ONC* ^a^ | *q*^2 b^ | *E*_s_ ^c^ | *r*^2 d^ | *F* ^e^ | RMSE ^f^ | | *P* ^g^ | *r*_p_^2 h^ | S ^i^ | E ^j^ | H ^k^ | D ^l^ | A ^m^ |
| CoMFA | 6 | 0.622 | 0.116 | 0.978 | 174.918 | 0.102 | 0.000 | | 0.933 | 0.677 | 0.323 | - | - | - |
| CoMSIA | 4 | 0.608 | 0.162 | 0.953 | 130.746 | 0.148 | 0.000 | | 0.914 | 0.353 | 0.221 | 0.300 | 0.115 | 0.011 |

^a^ Optimum number of components. ^b^ Leave-one-out (LOO) cross-validated correlation coefficient. ^c^ Standard error of estimate. ^d^ Noncross-validated correlation coefficient. ^e^ *F*-Test value. ^f^ Root mean squared error (RMSE). ^g^ Probability of *r*^2^. ^h^ Predicted correlation coefficient for the test set. ^i^ Steric field. ^j^ Electrostatic field. ^k^ Hydrophobic field. ^l^ H-bond donor field. ^m^ H-bond acceptor field.

**Table S2.** Structures, experimental, and predicted IC_50_ of the 3D-QSAR modeling data sets.

| Cmpd # | R Groups | X Group | IC_50_ ^a[23]^ (nM) | pIC_50_ | CoMFA | | |  | CoMSIA | | |
| --- | --- | --- | --- | --- | --- | --- | --- | --- | --- | --- | --- |
|  |  |  |  |  | Pred. ^b^ | Res. ^c^ | Error Rate |  | Pred. | Res. | Error Rate |
|  | **Training Set** | | | | | | | | | | |
| 290 | phenyl | H | 3.6 ± 0.8 | 8.444 | 8.359 | −0.085 | −1.01% |  | 8.234 | −0.210 | −2.49% |
| 291 | phenyl | 3-methyl | 6.7 ± 0.5 | 8.174 | 8.293 | 0.119 | 1.46% |  | 7.931 | −0.243 | −2.97% |
| 292 | phenyl | 5-methyl | 30 ± 18 | 7.523 | 7.461 | −0.062 | −0.82% |  | 7.510 | −0.013 | −0.17% |
| 293 | phenyl | 6-methyl | 22 ± 5 | 7.658 | 7.572 | −0.086 | −1.12% |  | 7.513 | −0.145 | −1.89% |
| 294 | phenyl | 3-fluoro | 4.9 ± 0.9 | 8.310 | 7.997 | −0.313 | −3.77% |  | 8.276 | −0.034 | −0.41% |
| 300 | pyridin-3-yl | H | 317.7 | 6.498 | 6.784 | 0.286 | 4.40% |  | 6.515 | 0.017 | 0.26% |
| 302 | 6-methoxypyridin-3-yl | H | 6.4 ± 1.7 | 8.194 | 8.231 | 0.037 | 0.45% |  | 8.077 | −0.117 | −1.43% |
| 303 | methyl | H | 317.7 | 6.498 | 6.552 | 0.054 | 0.83% |  | 6.746 | 0.248 | 3.82% |
| 304 | *tert*-butyl | H | 69 ± 30 | 7.161 | 7.224 | 0.063 | 0.88% |  | 7.316 | 0.155 | 2.16% |
| 306 | cyclohex-1-en-1-yl | H | 4.1 ± 1.3 | 8.387 | 8.339 | −0.048 | −0.57% |  | 8.330 | −0.057 | −0.68% |
| 309 | phenethyl | H | 6 ± 1.0 | 8.222 | 8.158 | −0.064 | −0.78% |  | 8.394 | 0.172 | 2.09% |
| 311 | 3-fluorophenyl | H | 3 ± 1.4 | 8.523 | 8.504 | −0.019 | −0.22% |  | 8.284 | −0.239 | −2.80% |
| 312 | 4-fluorophenyl | H | 2.9 ± 1.1 | 8.538 | 8.414 | −0.124 | −1.45% |  | 8.647 | 0.109 | 1.28% |
| 313 | 2,6-difluorophenyl | H | 4.8 ± 1.0 | 8.319 | 8.265 | −0.054 | −0.65% |  | 8.309 | −0.010 | −0.12% |
| 314 | 3,5-difluorophenyl | H | 3.9 ± 1.7 | 8.409 | 8.309 | −0.100 | −1.19% |  | 8.568 | 0.159 | 1.89% |
| 315 | 2,5-difluorophenyl | H | 4.4 ± 2.3 | 8.357 | 8.331 | −0.026 | −0.31% |  | 8.289 | −0.068 | −0.81% |
| 317 | 3,5-dimethylphenyl | H | 5.3 ± 1.9 | 8.276 | 8.346 | 0.070 | 0.85% |  | 8.631 | 0.355 | 4.29% |
| 318 | 2-methoxyphenyl | H | 12 ± 2 | 7.921 | 7.875 | −0.046 | −0.58% |  | 8.007 | 0.086 | 1.09% |
| 319 | 3-methoxyphenyl | H | 3.7 ± 1.2 | 8.432 | 8.415 | −0.017 | −0.20% |  | 8.360 | −0.072 | −0.85% |
| 320 | 4-methoxyphenyl | H | 2.6 ± 0.8 | 8.585 | 8.630 | 0.045 | 0.52% |  | 8.408 | −0.177 | −2.06% |
| 324 | 4-chloro-2-fluorophenyl | H | 1.0 ± 0.6 | 9.000 | 9.124 | 0.124 | 1.38% |  | 8.871 | −0.129 | −1.43% |
| 325 | 2,3,4-trifluorophenyl | H | 1.5 ± 0.2 | 8.824 | 8.910 | 0.086 | 0.97% |  | 8.828 | 0.004 | 0.05% |
| 326 | 4-chloro-2,3-difluorophenyl | H | 0.52 ± 0.39 | 9.284 | 9.424 | 0.140 | 1.51% |  | 9.229 | −0.055 | −0.59% |
| 327 | naphthalene-2-yl | H | 1.2 | 8.921 | 8.950 | 0.029 | 0.33% |  | 8.954 | 0.033 | 0.37% |
| 328 | 4-(pyridine-2-yl)phenyl | H | 1.3 ± 0.6 | 8.886 | 8.971 | 0.050 | 0.56% |  | 9.129 | 0.208 | 2.34% |
| 329 | 4-(pyridine-4-yl)phenyl | H | 1.2 ± 0.2 | 8.921 | 8.936 | 0.015 | 0.17% |  | 9.184 | 0.263 | 2.95% |
| 330 | 4-(2-methoxypyrimidin-5-yl)phenyl | H | 2.0 ± 1.0 | 8.699 | 8.661 | −0.038 | −0.44% |  | 8.666 | −0.033 | −0.38% |
| 331 | 4-(5-methoxypyrimidin-2-yl)phenyl | H | 1.2 ± 0.1 | 8.921 | 8.874 | −0.047 | −0.53% |  | 8.825 | −0.096 | −1.08% |
| 332 | 4-(thiazol-2-yl)phenyl | H | 1.1 ± 0.6 | 8.959 | 8.927 | −0.032 | −0.36% |  | 8.941 | −0.018 | −0.20% |
| 333 | 4-(isoxazol-3-yl)phenyl | H | 1.1 ± 0.3 | 8.959 | 8.932 | −0.027 | −0.30% |  | 8.967 | 0.008 | 0.09% |
| 334 | 4-(2*H*-1,2,3-triazol-2-yl)phenyl | H | 0.53 ± 0.12 | 9.276 | 9.344 | 0.068 | 0.73% |  | 9.174 | −0.102 | −1.10% |
|  | **Test Set** | | | | | | | | | | |
| 301 | pyridine-4-yl | H | 39 ± 8 | 7.409 | 7.807 | 0.398 | 5.37% |  | 7.484 | 0.075 | 1.01% |
| 305 | Cyclohexyl | H | 11 ± 3 | 7.959 | 7.789 | −0.170 | −2.14% |  | 8.010 | 0.051 | 0.64% |
| 308 | *E*-styryl | H | 3.2 ± 0.7 | 8.495 | 8.267 | −0.228 | −2.68% |  | 8.583 | 0.088 | 1.04% |
| 310 | 2-fluorophenyl | H | 2.2 ± 0.6 | 8.658 | 8.188 | −0.470 | −5.43% |  | 8.114 | −0.544 | −6.28% |
| 321 | 2-fluoro-3-methoxyphenyl | H | 1.8 ± 0.2 | 8.745 | 8.592 | −0.153 | −1.75% |  | 8.492 | −0.252 | −2.88% |
| 322 | 2-fluoro-4-methoxyphenyl | H | 1.1 ± 0.5 | 8.959 | 8.962 | 0.003 | 0.03% |  | 8.371 | −0.588 | −6.56% |
| 323 | 3-fluoro-4-methoxyphenyl | H | 2.0 ± 1.0 | 8.699 | 8.983 | 0.284 | 3.26% |  | 8.637 | −0.062 | −0.71% |





^a^ Enzyme inhibition test using *Pseudomonas aeruginosa* LpxC. ^b^ Pred. (Predicted values) stands for predicted pIC_50_ values. ^c^ Res. (Residual values) stands for residual values between experimental and predicted pIC_50_. IC_50_: half maximal inhibitory concentration.


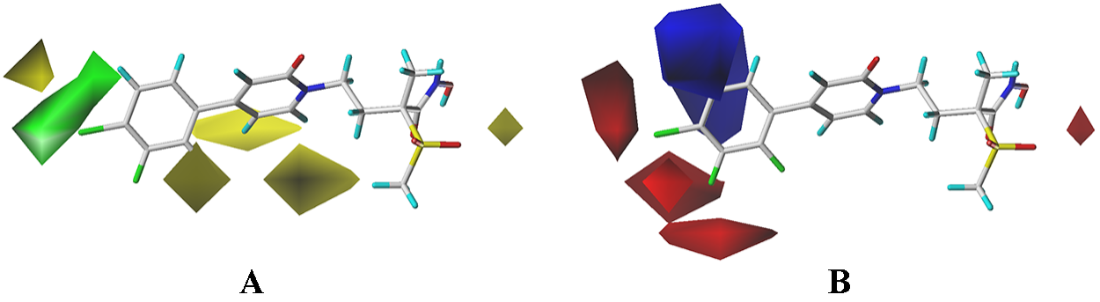


**Figure S1.** CoMFA contour maps for pyridone methylsulfone hydroxamate (PMH) compounds were represented in relation to Cmpd # 326. Steric (**A**): green contour map highlights the regions where bulky groups were beneficial for augmenting activity; yellow contours indicate the disfavored regions for the substitution of bulky groups. Electrostatic (**B**): blue contour map indicates where positively charged groups were beneficial for bioactivity, whereas red ones indicate where negatively charged groups were more favorable.


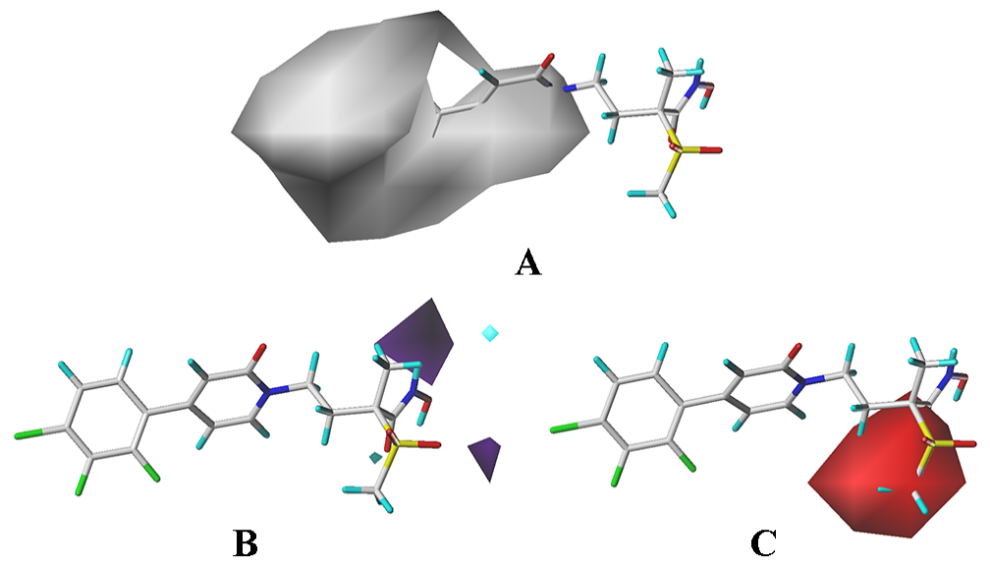


**Figure S2.** CoMSIA contour maps for PMH compounds were represented in relation to Cmpd # 326. Hydrophobic (**A**): the white contour indicates hydrophobic groups disfavored regions. Hydrogen bond donor (**B**): cyan contour maps indicate the favored regions of hydrogen bond donor; however purple ones indicate the disfavored region. Hydrogen bond acceptor (**C**): red contour presents the hydrogen bond acceptor disfavored region.

**Table S3.** Summary of the Mulliken charges analysis.

| **Cmpd # 290** | | **Cmpd # 318** | | **Cmpd # 326** | |
| --- | --- | --- | --- | --- | --- |
| Atoms | Mulliken Charge | Atoms | Mulliken Charge | Atoms | Mulliken Charge |
| N1 | −0.495350 | C1 | 0.175640 | C1 | 0.182679 |
| C2 | −0.127973 | C2 | −0.180358 | C2 | −0.190446 |
| C3 | 0.066432 | C3 | 0.167991 | C3 | 0.160757 |
| C4 | −0.134597 | N4 | −0.536855 | N4 | −0.525653 |
| C5 | 0.114325 | C5 | 0.605651 | C5 | 0.610269 |
| C6 | −0.178413 | C6 | −0.219629 | C6 | −0.211719 |
| C7 | 0.606228 | O7 | −0.622861 | O7 | −0.614762 |
| C8 | −0.174022 | C8 | −0.044077 | C8 | −0.065207 |
| C9 | 0.663178 | C9 | −0.195929 | C9 | −0.195227 |
| C10 | −0.382853 | C10 | −0.328284 | C10 | −0.309472 |
| C11 | −0.098765 | C11 | −0.298362 | C11 | −0.308346 |
| C12 | −0.095455 | C12 | 0.608771 | C12 | 0.587604 |
| C13 | −0.098506 | N13 | −0.260056 | N13 | −0.261376 |
| C14 | 0.140504 | H14 | 0.327053 | H14 | 0.321824 |
| C15 | −0.229954 | O15 | −0.566780 | O15 | −0.541398 |
| O16 | −0.603892 | O16 | −0.430184 | O16 | −0.396270 |
| C17 | −0.093729 | H17 | 0.348865 | H17 | 0.367842 |
| C18 | −0.300276 | S18 | 1.131648 | S18 | 1.107887 |
| S19 | 1.127670 | O19 | −0.611679 | O19 | −0.565175 |
| C20 | −0.525121 | O20 | −0.545059 | O20 | −0.576213 |
| O21 | −0.563815 | C21 | −0.501502 | C21 | −0.518914 |
| O22 | −0.567872 | C22 | 0.036640 | C22 | 0.035483 |
| O23 | −0.545523 | C23 | 0.301692 | C23 | 0.263040 |
| N24 | −0.306569 | C24 | −0.107939 | C24 | 0.340623 |
| O25 | −0.405818 | C25 | −0.068599 | F25 | −0.272927 |
| H26 | 0.104790 | C26 | −0.084524 | C26 | −0.177390 |
| H27 | 0.106176 | C27 | −0.111101 | F27 | −0.273414 |
| H28 | 0.118178 | O28 | −0.573729 | C28 | −0.047154 |
| H29 | 0.136751 | C29 | −0.074441 | Cl29 | 0.006168 |
| H30 | 0.151680 | H30 | 0.087620 | C30 | −0.101219 |
| H31 | 0.147314 | H31 | 0.142226 | H31 | 0.095828 |
| H32 | 0.156862 | H32 | 0.072617 | H32 | 0.137643 |
| H33 | 0.159663 | H33 | 0.159739 | H33 | 0.082781 |
| H34 | 0.108596 | H34 | 0.139206 | H34 | 0.164625 |
| H35 | 0.100418 | H35 | 0.159234 | H35 | 0.150137 |
| H36 | 0.110694 | H36 | 0.148832 | H36 | 0.158819 |
| H37 | 0.159975 | H37 | 0.139575 | H37 | 0.158210 |
| H38 | 0.109691 | H38 | 0.146753 | H38 | 0.156057 |
| H39 | 0.148923 | H39 | 0.153801 | H39 | 0.148513 |
| H40 | 0.148181 | H40 | 0.204490 | H40 | 0.135791 |
| H41 | 0.187338 | H41 | 0.189257 | H41 | 0.190972 |
| H42 | 0.190962 | H42 | 0.184424 | H42 | 0.194042 |
| H43 | 0.175104 | H43 | 0.086656 | H43 | 0.166997 |
| H44 | 0.327096 | H44 | 0.090390 | H44 | 0.118009 |
| H45 | 0.361771 | H45 | 0.084045 | H45 | 0.109682 |
| NA | - | H46 | 0.086897 | NA | - |
| NA | - | H47 | 0.132616 | NA | - |
| NA | - | H48 | 0.124781 | NA | - |
| NA | − | H49 | 0.124837 | NA | − |





**Figure S3.** Correlation between the mean binding energy calculated under the AutoDock4_Zn_ force field of the largest conformational cluster of the PMH UDP-3-O-(R-3-hydroxymyristoyl)-N-acetylglucosamine deacetylase (LpxC) inhibitors and pIC_50_ measured by the experiment.

**Table S4.** Hydrogen bonds between Cmpd # 290 and *Pseudomonas aeruginosa* LpxC (PaLpxC).

| **Donor** | **Acceptor** | **AvgDist.^a^ (Å)** | **AvgAng.^b^ (°)** | **Frec.^c^ (%)** |
| --- | --- | --- | --- | --- |
| Cmpd # 290-N2-H90 | M62-O | 2.94 ±0.01 | 161.44 ± 8.83 | 97.49 |
| T190-OG1-HG1 | Cmpd # 290-O4 | 2.76 ± 0.01 | 155.52 ± 7.74 | 94.07 |
| H264-NE2-HE2 | Cmpd # 290-O5 | 2.79 ± 0.01 | 154.76 ± 7.66 | 87.22 |
| H264-NE2-HE2 | Cmpd # 290-N2 | 3.28 ± 0.01 | 149.84 ± 9.46 | 65.15 |
| K238-NZ-HZ3 | Cmpd # 290-O3 | 3.05 ± 0.01 | 146.69 ± 10.33 | 8.13 |
| K238-NZ-HZ2 | Cmpd # 290-O3 | 3.05 ± 0.01 | 146.48 ± 10.69 | 8.01 |
| K238-NZ-HZ1 | Cmpd # 290-O3 | 3.06 ± 0.01 | 146.55 ± 10.25 | 6.64 |

^a^ AvgDist. stands for the average distance between the H-bond donor atom and the acceptor atom. ^b^ AvgAng. stands for the average angle of the donor atom, hydrogen atom, and acceptor atom. ^c^ Frac. is the fraction of frames where the H-bond is present in MD simulation.

**Table S5.** Contribution to binding free energy by each kind of energy (kcal/mol).

| **Items** | **PaLpxC** | **Cmpd # 290** | **Complex** | **Delta** |
| --- | --- | --- | --- | --- |
| *ELE*_IN_ ^a^ | −9653.58 ± 79.55 | 16.89 ± 2.47 | −9636.45 ± 76.86 | 0.24 |
| *VDW*_IN_ ^b^ | −1276.38 ± 32.89 | 9.63 ± 1.80 | −1310.92 ± 31.60 | −44.16 |
| *ELE*_PB_ ^c^ | −3392.06 ± 55.78 | −45.53 ± 1.66 | −3420.35 ± 53.82 | 17.24 |
| *VDW*_PB_ ^d^ | 76.39 ± 0.78 | 4.12 ± 0.02 | 75.09 ± 0.72 | −5.42 |
| *H* | −7280.90 ± 39.98 | 31.82 ± 4.97 | −7281.18 ± 39.06 | −32.10 |
| *TS* | 3313.34 ± 6.33 | 50.63 ± 0.05 | 3345.65 ± 5.48 | −18.31 |
| Δ*G*_binding_ | −13.79 | | | |

^a^ *ELE*_IN_ stands for the sum of non-bonded electrostatic energy and 1,4-electrostatic energy. ^b^ *VDW*_IN_ stands for the sum of non-bonded van der Waals energy and 1,4-van der Waals energy. ^c^ *ELE*_PB_ represents reaction field energy calculated by the Poisson-Boltzmann (PB) method. ^d^ *VDW*_PB_ represents the hydrophobic contribution to solvation free energy calculated by the PB method.

**Table S6.** Energy decomposition of the key residues in PaLpxC significantly concerned with binding ligand Cmpd # 290 (kcal/mol)

| **No.** | **Residue** | ***E*_VDW_ ^a^** | ***E*_ELE_ ^b^** | ***E*_GB_ ^c^** | ***E*_GBSUR_ ^d^** | ***E*_TOT_ ^e^** |
| --- | --- | --- | --- | --- | --- | --- |
| 1 | D241 | −0.39 ± 0.54 | 7.25 ± 1.29 | −8.80 ± 1.65 | −0.02 ± 0.01 | −1.97 ± 0.83 |
| 2 | F191 | −3.10 ± 0.41 | 0.65 ± 0.91 | 0.76 ± 0.79 | −0.20 ± 0.01 | −1.88 ± 0.45 |
| 3 | K238 | −0.48 ± 0.61 | −32.92 ± 3.75 | 31.69 ± 2.83 | −0.13 ± 0.02 | −1.84 ± 1.52 |
| 4 | M62 | −2.72 ± 0.50 | 1.06 ± 0.63 | 0.16 ± 0.55 | −0.30 ± 0.04 | −1.80 ± 0.69 |
| 5 | I197 | −1.53 ± 0.27 | −0.08 ± 0.07 | 0.15 ± 0.06 | −0.15 ± 0.03 | −1.60 ± 0.27 |
| 6 | T190 | −1.25 ± 0.61 | −2.69 ± 1.20 | 2.97 ± 1.17 | −0.15 ± 0.03 | −1.13 ± 0.58 |
| 7 | H264 | −1.10 ± 0.37 | −21.08 ± 2.39 | 21.13 ± 2.18 | −0.06 ± 0.01 | −1.11 ± 0.50 |
| 8 | G192 | −1.11 ± 0.28 | −0.65 ± 0.16 | 1.01 ± 0.16 | −0.09 ± 0.02 | −0.83 ± 0.27 |
| 9 | G209 | −0.80 ± 0.17 | −0.82 ± 0.23 | 0.89 ± 0.26 | −0.11 ± 0.01 | −0.83 ± 0.28 |
| 10 | L200 | −0.76 ± 0.19 | −0.38 ± 0.08 | 0.42 ± 0.07 | −0.06 ± 0.03 | −0.79 ± 0.21 |
| 11 | V216 | −0.51 ± 0.17 | −0.26 ± 0.08 | 0.19 ± 0.08 | −0.03 ± 0.01 | −0.61 ± 0.19 |
| 12 | H237 | −0.79 ± 0.19 | −2.39 ± 0.61 | 2.64 ± 0.41 | −0.02 ± 0.01 | −0.56 ± 0.32 |
| 13 | G263 | −0.26 ± 0.11 | −2.29 ± 0.64 | 2.03 ± 0.37 | −0.02 ± 0.01 | −0.54 ± 0.42 |

^a^ *E*_VDW_ stands for the change of van der Waals energy part due to total residual. ^b^ *E*_ELE_ stands for the change of electrostatic energy part due to total residual. ^c^ *E*_GB_ stands for the change of reaction field energy part due to residual calculated by generalized Born (GB) method. ^d^ *E*_GBSUR_ stands for the change of hydrophobic contribution to solvation free energy due to total residual calculated by GB method. ^e^ *E*_TOT_ stands for the change of total energy due to total residual.


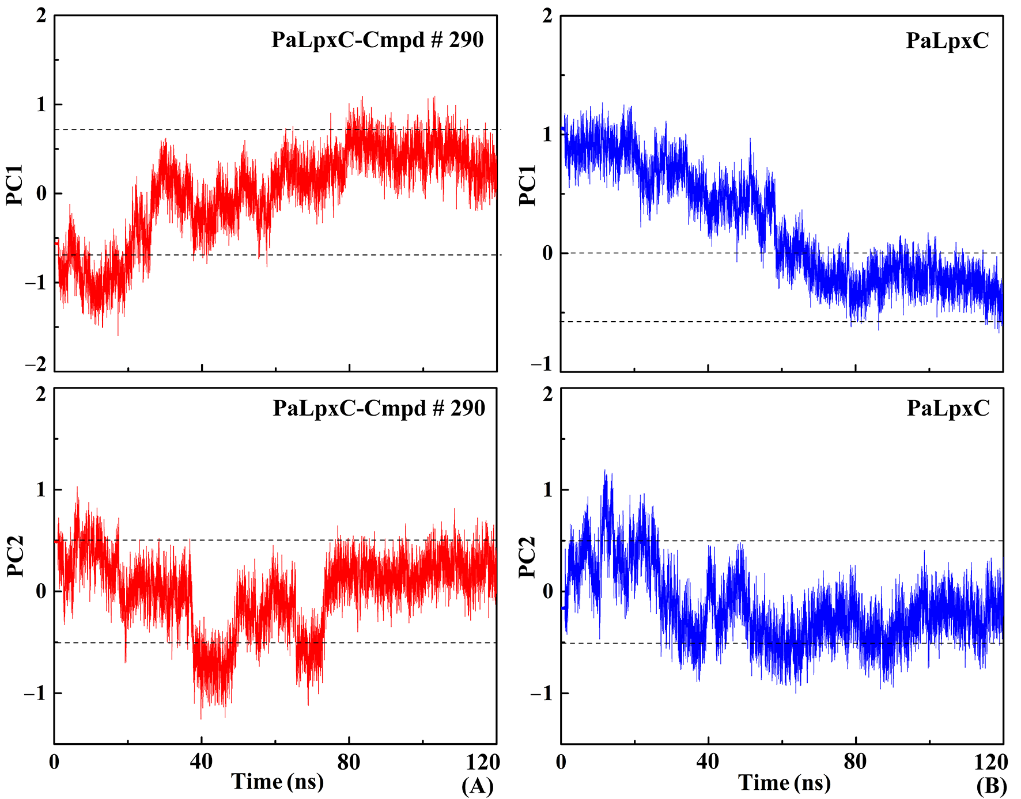


**Figure S4.** Principal component 1 (PC1) and principal component 2 (PC2) versus simulation time in the complex PaLpxC-Cmpd # 290 (A) and PaLpxC protein (B) systems.


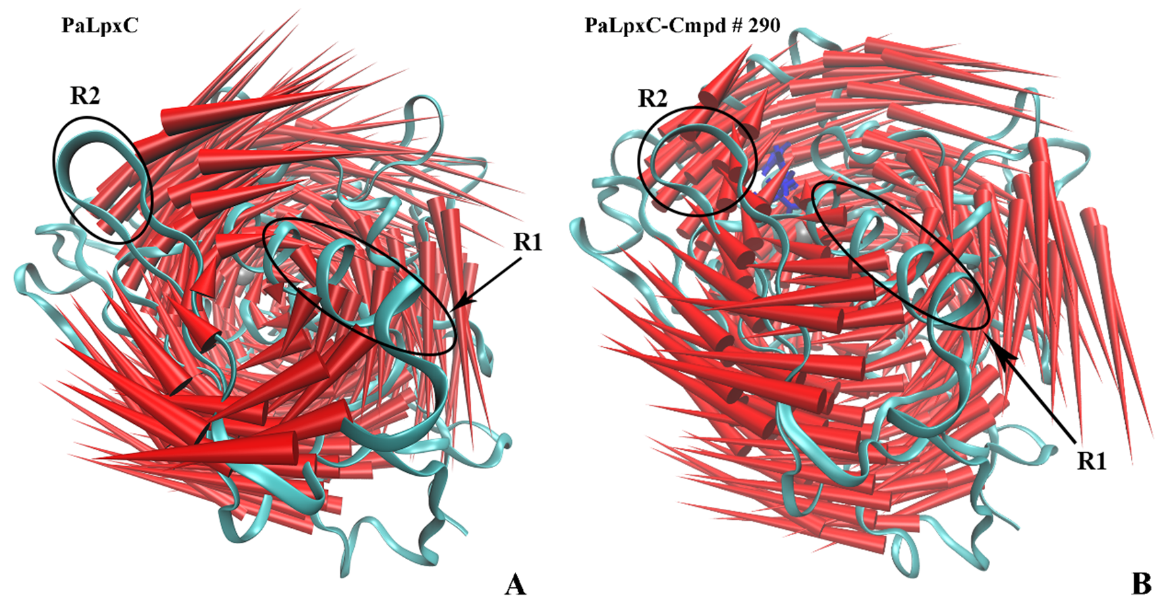


**Figure S5.** Slow motion modes of the PaLpxC (**A**) and PaLpxC-Cmpd # 290 (**B**) systems. R1 and R2 both are the specific regions experiencing obvious changes of motion direction between the two systems. The proteinic structure is displayed with the New Ribbon model, and the gray Corey-Pauling-Koltun (CPK) model and blue stick model represent the zinc ion and Cmpd # 290, respectively.
