# Supplementary material for: 3D-QSAR, Molecular Docking and Molecular Dynamics Simulation of Pseudomonas aeruginosa LpxC Inhibitors
Source: Int J Mol Sci. 2017 May 6;18(5):761. doi: 10.3390/ijms18050761 (PMC5454807; doi:10.3390/ijms18050761)
Supplement: Supplementary file 1 [file ijms-18-00761-s001.zip › ijms-180988-final.docx]

Article

3D-QSAR, Molecular Docking and Molecular Dynamics Simulation of *Pseudomonas* *aeruginosa* LpxC Inhibitors

Ke Zuo ^1,†^, Li Liang ^1,†^, Wenyi Du ^1^, Xin Sun ^1^, Wei Liu ^1^, Xiaojun Gou ^1,^*, Hua Wan ^2^ and
Jianping Hu ^1,3,^*

^1^ College of Pharmacy and Biological Engineering, Key Laboratory of Medicinal and Edible Plants Resources Development of Sichuan Education Department, Sichuan Industrial Institute of Antibiotics, Chengdu University, Chengdu 610106, China; zuoke2015@outlook.com (K.Z.); lianglicdu@163.com (L.L.); wydu2017@gmail.com (W.D.); sunbsxw123@163.com (X.S.); liuwei@cdu.edu.cn (L.W.)

^2^ College of Mathematics and Informatics, South China Agricultural University, Guangzhou 510642, China; wanhua@scau.edu.cn

^3^ College of Chemistry, Leshan Normal University, Leshan 614004, China

* Correspondence: gxjmeprd@163.com (X.G.); hujianping@emails.bjut.edu.cn (J.H.);
Tel.: +86-28-8461-6301 (X.G.); +86-28-6461-6301 (J.H.)

† These authors contributed equally to this work

Academic Editor: Lyle Isaacs

Received: 11 February 2017; Accepted: 26 March 2017; Published: xx March 2017

**Abstract:** As an important target for the development of novel antibiotics, UDP-3-*O*-(*R*-3-hydroxymyristoyl)-*N*-acetylglucosamine deacetylase (LpxC) has been widely studied. Pyridone methylsulfone hydroxamate (PMH) compounds can effectively inhibit the catalytic activity of LpxC. In this work, the three-dimensional quantitative structure-activity relationships of PMH inhibitors were explored and models with good predictive ability were established using comparative molecular field analysis and comparative molecular similarity index analysis methods. The effect of PMH inhibitors’ electrostatic potential on the inhibitory ability of *Pseudomonas* *aeruginosa* LpxC (PaLpxC) is revealed at the molecular level via molecular electrostatic potential analyses. Then, two molecular dynamics simulations for the PaLpxC and PaLpxC-inhibitor systems were also performed respectively to investigate the key residues of PaLpxC hydrolase binding to water molecules. The results indicate that orderly alternative water molecules can form stable hydrogen bonds with M62, E77, T190, and H264 in the catalytic center, and tetracoordinate to zinc ion along with H78, H237, and D241. It was found that the conformational transition space of PaLpxC changes after association with PMH inhibitors through free energy landscape and cluster analyses. Finally, a possible inhibitory mechanism of PMH inhibitors was proposed, based on our molecular simulation. This paper will provide a theoretical basis for the molecular design of LpxC-targeting antibiotics.

**Keywords:** drug-resistance; LpxC; methylsulfone hydroxamate; 3D-QSAR; molecular dynamics simulation; free energy landscape; inhibitory mechanism

1. Introduction

Bacterial drug-resistance has become a public health problem worldwide and a serious threat to human health [1,2]. Recently, bacteria with the multiple drug-resistant (MDR) mechanism, also called “superbugs”, have been reported all over the world [3–5], such as pan drug-resistant *Acinectobacter* *baumannii*, carbapenem-resistant *Klebsiella* *pneumoniae*, and *Pseudomonas* *aeruginosa*. Most of these “superbugs” are Gram-negative (G^−^) bacteria possessing complex cell membranes, which strongly obstructs antibacterial agents’ ability to penetrate into the cell. This makes the treatment of MDR bacterial infection much more difficult.

The main constituent of endotoxin, lipopolysaccharide (LPS), is found in the outer leaflet of the outer membrane of the cell wall, and is a special component of G^−^ bacteria, which can cause fever, microcirculatory disturbance, and even shock [6]. The assembly process of LPS on the cell wall is mainly mediated by lipid A (LA) [7], thus inhibiting the biosynthesis of LA may lead to deficiencies in the G^−^ bacterial cell wall, the increased vulnerability of G^−^ bacteria, and the propensity for cell death. Highly conserved UDP-3-*O*-(*R*-3-hydroxymyristoyl)-*N*-acetylglucosamine deacetylase (LpxC) among G^−^ bacteria (more than 40 species) is the first key enzyme in LA biosynthesis [8,9], and also shows no homology to mammalian and human proteins [10]. Accordingly, LpxC is an ideal new target for antibacterial drug design.

LpxC is a Zn^2+^-dependent enzyme. To date, 44 LpxC crystal structures from four strains are included in the RCSB [11] protein data bank. Among them, 42 crystal structures were obtained by X-ray diffraction experiments and the remaining two by nuclear magnetic resonance (NMR) experiments. From crystallographic studies, LpxC has a “β-α-α-β” sandwich structure, encompassing two similar topological domains (Domain I and Domain II). Each domain contains a helix layer consisting of two α-helixes and a sheet layer made up of five parallel or antiparallel β-strands [12,13]. Moreover, there are two unique amino acid fragments, Insert I and Insert II, interposing into Domain I and Domain II, respectively. Insert I comprises three β-sheets, which partially defines the range of active site and stabilizes the catalytic zinc ion; whereas Insert II is composed of a special “β-α-β” substructure, and forms a topologically closed hydrophobic channel [14,15].

Based on the enzyme structure, most of the LpxC inhibitors have been reported [16] to contain a hydroxamate acid moiety which can chelate to Zn^2+^ and a hydrophobic chain formed by the phenylalkyne moiety, so as to adapt to the hydrophobic channel of the enzyme. In the last two years, Ralph et al. designed and synthesized a series of glyceric acid derivatives and furanosidic derivatives, which showed good inhibitory activity against *Escherichia* *coli* LpxC (EcLpxC) [17–19]. Kurasaki et al. designed, synthesized, and evaluated oxazolidinone derivatives through the scaffold hopping method, which would strongly inhibit wild type EcLpxC [20]. The Lemaitre group reported types of biphenyl-diacetylene-based difluoromethyl-allo-threonyl-hydroxamate LpxC inhibitors possessing high inhibitory activity against four MDR strains [21]. Abdel-Magid also designed six 1,2-dihydro-3*H*-pyrrolo[1,2-c]imidazol-3-one derivatives as LpxC inhibitors, and these compounds were able to effectively inhibit partial strains of *Escherichia* *coli*, *Pseudomonas* *aeruginosa*, and *Klebsiella* *pneumoniae* [22]. Furthermore, Yang et al. also reported two kinds of compounds containing kojic acid derivative structures and a methylsulfone moiety at the hydrophilic terminus [23]. Results from pharmacokinetic experiments indicated that the methylsulfone moiety might serve as the dominant group of LpxC inhibitors. Because the antibacterial mechanism of the LpxC inhibitor is different from those of the existing antibacterial agents, it exhibits a better inhibitory activity on the current MDR bacteria. Montgomery et al. [24] reported a series of pyridine methylsulfone hydroxamate (PMH) LpxC inhibiors, exhibiting strong inhibitory activity against *Pseudomonas* *aeruginosa*, *Escherichia* *coli*, and other G^−^ bacteria. Thus, PMH LpxC inhibitors are potential new antimicrobial agents.

There are a lot of studies on the species diversity of LpxC, catalytic mechanism, and the design and synthesis of LpxC inhibitors. Nevertheless, three key scientific issues of PMH LpxC inhibitors (i.e., molecular recognition, inhibitory mechanism, and the motion pattern with LpxC) have not been reported in detail. In this work, we first integrate the literature report from the existing database ChEMBL [25] to establish a virtual library of LpxC inhibitors, then select 38 PMH compounds (Cmpd # 290–294, 300–306, 308–315, 317–334) with a pyridone parent ring and relatively uniform activity distribution to establish three-dimensional quantitative structure-activity relationships (3D-QSAR) models. To explore the possible inhibitory mechanism of PMH LpxC inhibitors, molecular dynamics (MD) simulations for *Pseudomonas aeruginosa* LpxC (PaLpxC) and PaLpxC-inhibitor systems were performed comparatively. The difference of the motion patterns between PaLpxC and its complex with inhibitors were investigated using conformational cluster and free energy landscape (FEL) analyses (see Figure 1). These studies will provide a theoretical basis for the activity prediction, molecular design, and modification of PMH LpxC inhibitors.


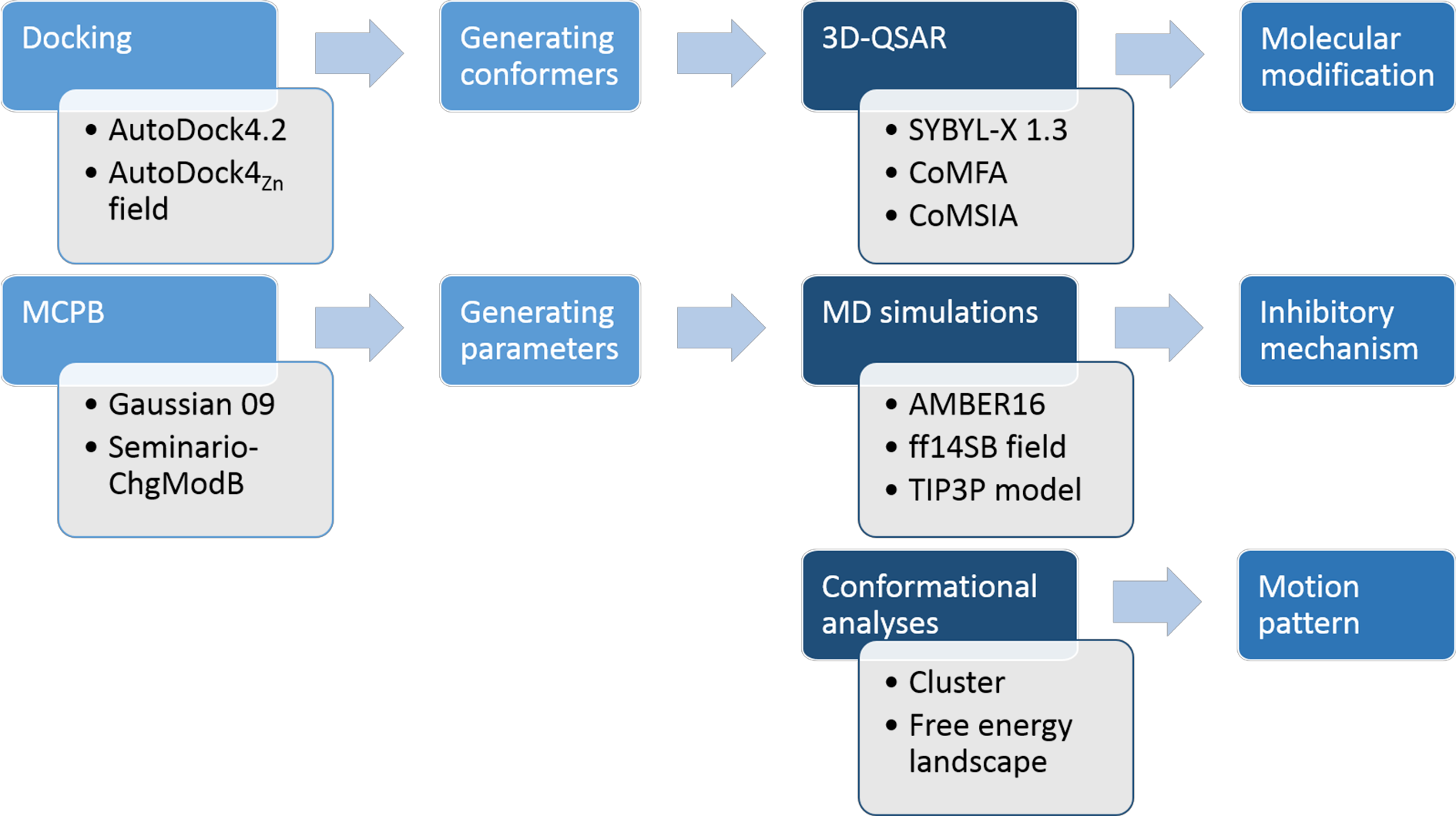


**Figure 1.** Protocol of this work. 3D-QSAR: three-dimensional quantitative structure-activity relationships; CoMFA: comparative molecular field analysis; CoMSIA: comparative molecular similarity index analysis; MD: molecular dynamics.

2. Results and Discussion

2.1. Systems for Simulation

PMH LpxC inhibitors belong to a group of traditional hydroxamate molecules, which mainly suppress the activity of zinc ions at the bottom of LpxC's active pocket relying on the hydrophilic terminal hydroxamate moiety [10,14–22,24]. Figure 2 shows the binding mode of Cmpd # 290 with PaLpxC and the molecular alignment of the PMH LpxC inhibitors. It is worth mentioning that the binding details will be analyzed below (see section on “molecular docking”). As shown in Figure 2, the public substructure of PMH molecules (i.e., pyridone methylsulfone hydroxamate) is aligned well, which maximizes the similarity with the spatial orientation of the molecules, and provides a good foundation for the subsequent generation of the comparative molecular field analysis (CoMFA) and comparative molecular similarity index analysis (CoMSIA) models.


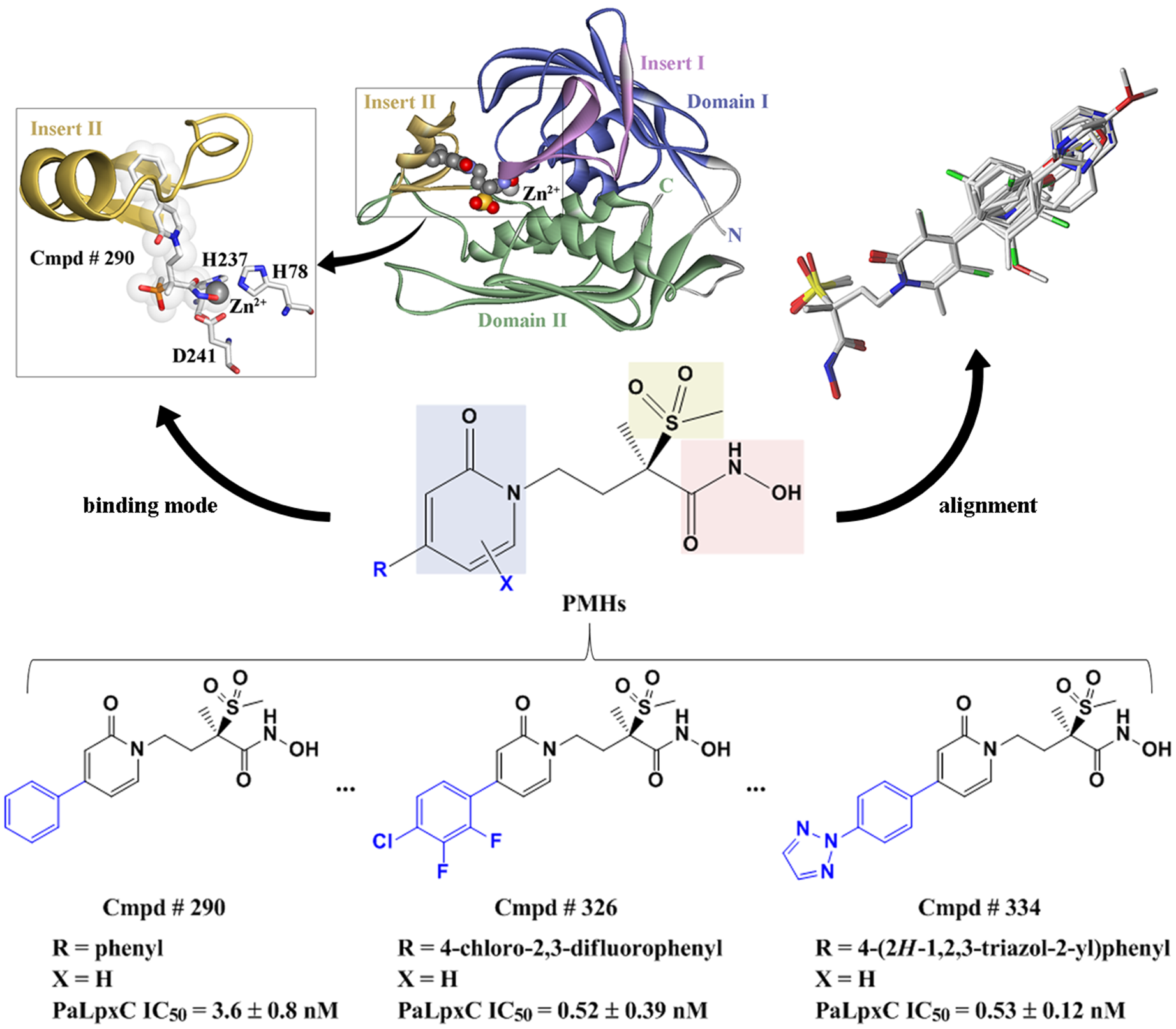


**Figure 2.** Structural alignment of pyridone methylsulfone hydroxamate compounds for the generation of 3D-QSAR models and its binding mode at the *Pseudomonas aeruginosa* LpxC (PaLpxC) active site. Compound (Cmpd) # 290, Cmpd # 326**,** and Cmpd # 334 are the representatives of pyridone methylsulfone hydroxamate (PMH) compounds in the virtual database of LpxC inhibitors. IC_50_: half maximal inhibitory concentration.

2.2. CoMFA and CoMSIA Models

In this work, 31 PMH LpxC inhibitors (training set) were used for the establishment of the 3D-QSAR models, with the related parameters and results shown in Table S1. In the CoMFA model, the cross-validated correlation coefficient (*q*^2^) and noncross-validated correlation coefficient (*r*^2^) are 0.622 and 0.978, respectively. The root mean squared error (RMSE) is 0.102, and the combination with the high predicted correlation coefficient (*r*2 p = 0.933) confirms the reasonability and reliability of this model. According to the CoMFA model, the contribution of the steric field (S) is 67.7%, and the electrostatic field (E) is 32.3%. The model indicates that the steric field surrounding the PMH LpxC inhibitors plays an important role in its inhibitory activity. The CoMSIA model also analyzes the hydrophobic field (H), hydrogen bond (H-bond) acceptor field (A), and H-bond donor field (D) of the training set molecules beyond the steric field and electrostatic field. In light of the CoMSIA model, the contribution of S is 35.3%, while that of E is 22.1%. Moreover, the hydrophobic filed portion occupies 30.0%, and the H-bond donor field and acceptor field hold 11.5% and 1.1%, respectively. The steric and hydrophobic fields of PMH LpxC inhibitors were shown to contribute greatly to their biological activities, followed by the electrostatic field and H-bond field. Based on the results of the CoMFA and CoMSIA models, it is speculated that changing the bulk and hydrophobicity of the molecules may be an important method to improve the biological activity of PMH LpxC inhibitors.

Figure 3 displays the correlation of predicting the pIC_50_ values and experimental ones of PMH LpxC inhibitors between the CoMFA model (A) and CoMSIA model (B), respectively. As seen from Figure 3, there is a significant linear correlation between the predicted pIC_50_ and the experimental values, which proves the reliability of the two models.


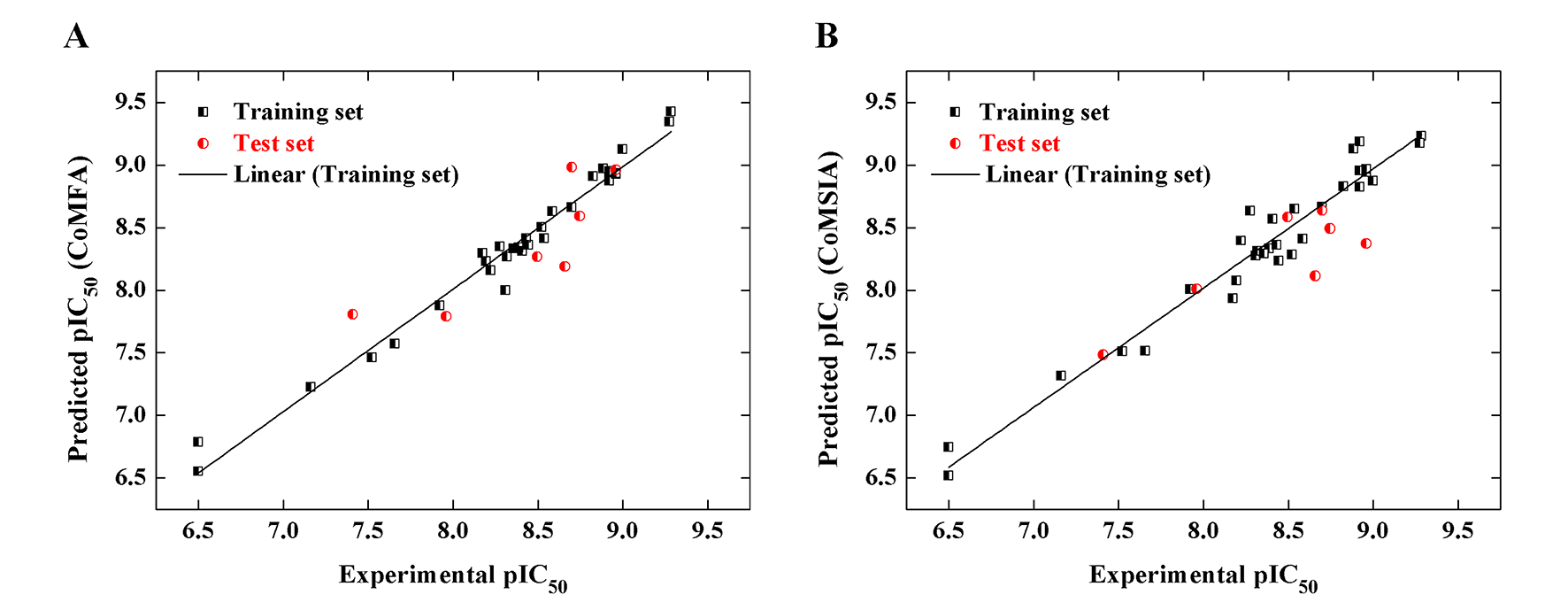


**Figure 3.** Correlation between experimental and predicted pIC_50_ values for training (black) and test (red) set compounds based on the comparative molecular field analysis (CoMFA) model (**A**); and comparative molecular similarity index analysis (CoMSIA) model (**B**).

Table S2 lists the predicted activity of molecules in the test set based on the established CoMFA model and CoMSIA model. The deviation of negative logarithm form of IC_50_ (pIC_50_) between the predicted and experimental values is less than 1, and some individual results are very consistent with the experimental values, such as Cmpd # 322. The results show that the CoMFA model established from this training set has good stability and predictive ability for the inhibitory activity of PMH LpxC molecules. By comparing the prediction results of the CoMSIA model with the CoMFA model, it is found that the overall predictive ability of the CoMSIA model is slightly inferior to that of the CoMFA model. Nevertheless, the CoMSIA model still has good predictive ability and high stability, and can complement the CoMFA model. The former provides more comprehensive information for drug design based on 3D-QSAR, as well as for the modification of the lead compounds.

2.3. CoMFA and CoMSIA Contour Maps

Figure S1 shows the contour maps of the CoMFA model for Cmpd # 326 with the highest activity; the truncation ratio of the contour line is 80%:20%. It can be seen from the steric field contour map (see Figure S1A) around the PMH LpxC inhibitor that there are a total of five yellow and one green contours. The yellow contours are mainly distributed in the hydrophobic side of the molecule and only one block is located in the methylsulfone terminus, suggesting that the introduction of large volume groups is not conducive to the increase of inhibitory activity. For example, the inhibitory activity of Cmpd # 292 with the introduction of methyl in the para-position of the pyridone ring is significantly lower than that of non-substituted Cmpd # 290. The green blocks at the end of the hydrophobic chain suggest that introducing large volume groups in that region will increase the inhibitory activity of the PMH LpxC inhibitors, which explains the higher inhibitory activity of Cmpds # 328–334 relative to Cmpd # 290 (see Table S2). However, in the electrostatic field contour map (see Figure S1B), there are a total of four red and one blue contours at the end of the phenyl ring, which indicate that an increase of the positive and negative charges in these regions are beneficial to improving inhibitory activity. Taking Cmpd # 326 and # 290 as an example, the introduction of two F atoms and one Cl atom with strong electron-withdrawing effects into the phenyl ring notably increases the electron cloud density. Therefore, the inhibitory activity of Cmpd # 326 is significantly higher than that of Cmpd # 290 (see Table S2). In addition, at the methylsulfone terminus, there is also a red contour, suggesting that increasing the number of negative charges of the methylsulfone moiety may enhance the inhibitory activity of the PMH LpxC inhibitors.

The CoMSIA model can show five S, H, E, A, and D different molecular field results, which can provide more information for molecular design than the CoMFA model. Figure S2A–C, respectively, shows the hydrophobic field, H-bond acceptor field, and H-bond donor field contour maps of the CoMSIA model. The reference molecule in the drawing is Cmpd # 326. In Figure S2A, we can see that almost all of the hydrophobic chains are wrapped by grey contours, which indicates that the introduction of hydrophobic groups will not be conducive to the increase of molecular biological activity. In Figure S2B, cyan and purple contour maps represent the favored and disfavored regions of the H-bond donor, respectively. In Figure S2C, the red contour maps represent the disfavored regions of the H-bond acceptor. The H-bond is one of the important non-bonding interactions between drug molecules and receptors. According to these contour maps, the introduction of more H-bond donors at the oxime and the methylsulfone groups is unexpectedly not conducive to enhancing the activity of PMH LpxC inhibitors. Similarly, the introduction of more H-bonds acceptors at the methylsulfone substituent is also not conducive to the improvement of the activity of these inhibitors theoretically.

In general, the CoMFA and CoMSIA models established in this work have good predictive ability. Additionally, the CoMFA and CoMSIA contour maps both also visually exhibit the relationship between the molecular field properties (including substituent groups, charge distribution, hydrophobicity, number of H-bond donor and acceptor atoms) and inhibitory activity of PMH LpxC inhibitors. On the basis of the contour maps, the introduction of bulky groups at the para-position of the carbonyl on the pyridone ring will enhance the negative charge of the phenyl terminus and also hinder the introduction of highly hydrophobic groups. Similarly, the avoidance of substituents containing H-bond donor or acceptor atoms near the end of the hydroxamate moiety may improve the inhibitory activity of PMH LpxC inhibitors.

2.4. MESP and Mulliken Charge Analyses

The strength of the interaction between the inhibitor molecule and the specific target is usually dependent on the unique structure and charge properties of the inhibitor molecule itself. Through calculation and analysis of the charge properties of inhibitors, it is helpful to investigate the relationship between the charge distribution and the inhibitory activity. In this work, Cmpd # 318, Cmpd # 290, and Cmpd # 326, which have similar molecular weights but different inhibitory activities, were chosen for molecular electrostatic potential (MESP) and Mulliken charge analyses. The results of the Mulliken charge calculation are listed in Table S3.

Figure 4 shows the MESP distribution maps of Cmpd # 290 (A), Cmpd # 318 (B), and Cmpd # 326 (C). The red part indicates the region of the most negative charge, which shows the strongest electronic donation region. The blue part represents the region of the most positive charge, meaning the strongest electronic acceptance region. As can be seen from Figure 4, the negative charge region of Cmpd # 290 is mainly concentrated in the carbonyl oxygen of the pyridone ring and the oxygen of methylsulfone, suggesting these areas are H-bond acceptor regions; however, the positive charge region is mainly concentrated in the vicinity of the hydroximate moiety, which can coordinate with the lone electron pair of Zn^2+^, and inhibit its catalytic activity. Cmpd # 318 is obtained by introducing a methoxy group into the 2-position of the phenyl moiety in Cmpd # 290 (see Figure 4B). The resonance donating effect of methoxy increases the negative charge at the para-position of the conjugated system; while the negative charge at sulfone and the positive charge of the hydroxamate group decreases. Sulfone and hydroxamate both are the key groups of PMH LpxC inhibitors, and the change of the charge properties might be an important factor for the lower inhibitory activity of Cmpd # 318 compared to that of Cmpd # 290. Moreover, the hydrogen atoms at the 2,3-positon on the phenyl group of Cmpd # 290 are replaced by fluorine atoms. With the chlorine atom introduced into the 4-position of the phenyl group, Cmpd # 326 is obtained. The MESP distribution of Cmpd # 326 is shown in Figure 4C. Comparing it to Figure 4A, the halogen atoms have little effect on the charge properties of the above three sites, resulting in only a slight decrease. However, the polarization of the electrons in the molecular conjugate system changes obviously, which is beneficial to the enhancement of inhibitory activity. Thus, it is speculated that the increase of the inhibitory activity of Cmpd # 326 is related to the electrostatic potential change of the hydrophobic terminus.

**
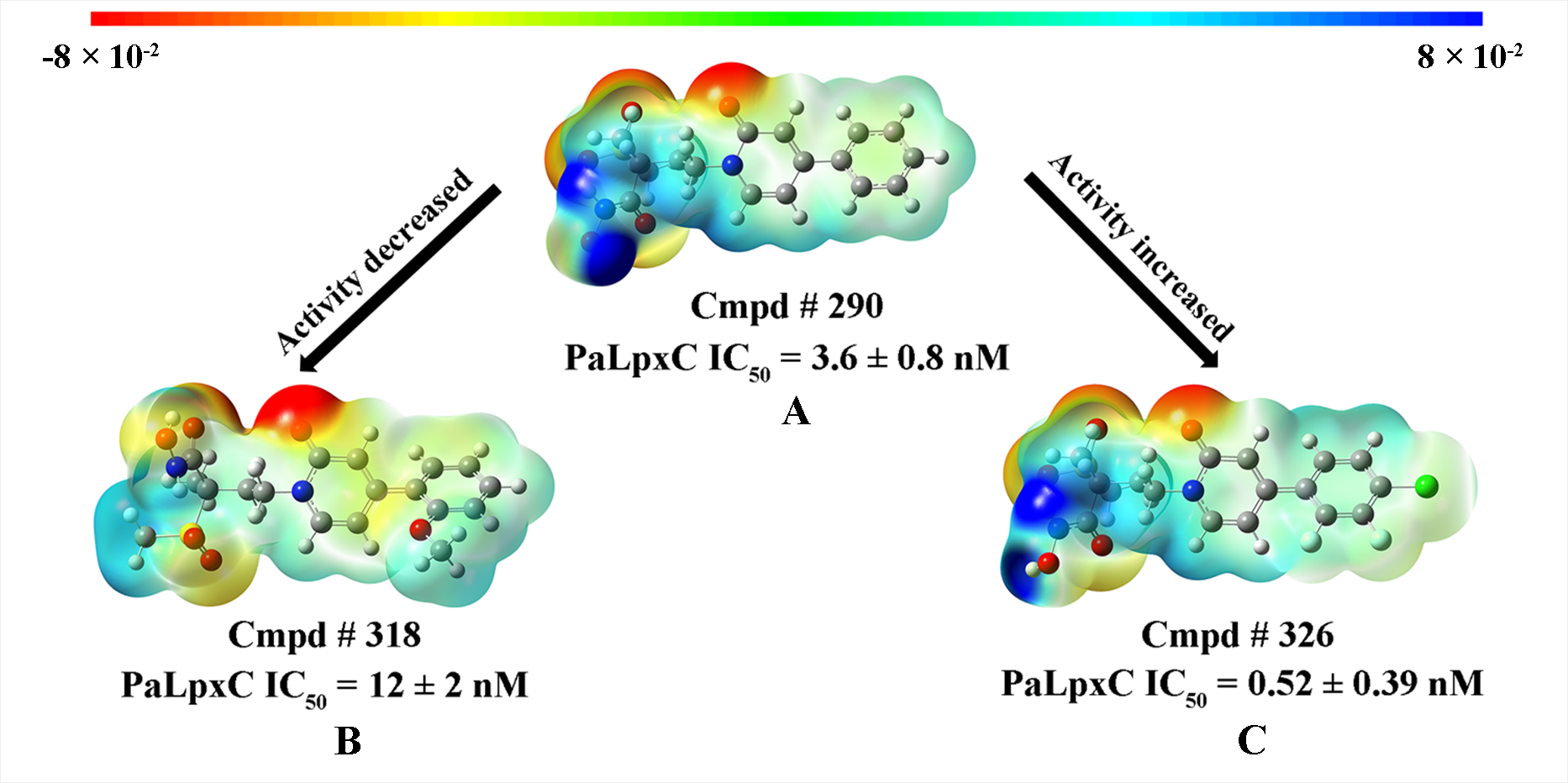
**

**Figure 4.** Molecular electrostatic potential map superimposed onto a surface of constant electron density for PMH LpxC inhibitors Cmpd # 290 (**A**); Cmpd # 318 (**B**); and Cmpd # 326 (**C**). Red/blue color respectively indicates the highly electronegative/electropositive regions, while the regions with the other colors represent the potential between the two strongly electronegative and electropositive regions.

2.5. Molecular Docking

Molecular docking was performed with the AutoDock4_Zn_ force field, and the results are shown in Figure 5. As can be seen in Figure 5A, the 38 PMH LpxC inhibitors are well bound in the active pocket. The binding mode of the docking result is consistent with that of the co-crystal structure (3UHM.pdb): the hydroxamate moiety coordinates with Zn^2+^, and the phenyl pyridone chain is well combined in the hydrophobic channel of PaLpxC. Figure 5B shows the interactions between the inhibitor Cmpd # 290 and PaLpxC in the co-crystal structure. The purple dashed lines in the figure represent the hydrophobic interaction, which contains an amide-π packing interaction between the pyridone ring and G192, π-alkyl mixed hydrophobic interactions between F191 and the methyl of Cmpd # 290, and the π-σ mixed hydrophobic interactions formed between phenyl pyridone and L18, A206, A214, and V216. The green dashed lines indicate the H-bonds, which mainly exist between the sulfone oxygen atoms and the ω-amino group of K238, and between the β-hydroxyl of T190 and the carbonyl oxygen of the hydroxamate moiety. In addition, it is also observed that there is a certain electrostatic interaction between Zn^2+^ and the carboxyl group of E77 (red dashed lines).

Figure S3 shows the correlation between the calculated average binding free energies and the experimental pIC_50_ values of the PMH LpxC inhibitors. As can be seen from Figure S3, the correlation coefficient (*r*^2^) between them is 0.312. To some extent, the molecular docking method based on the AutoDock4_Zn_ force field can be used to predict the biological activity of PMH LpxC
inhibitors roughly.


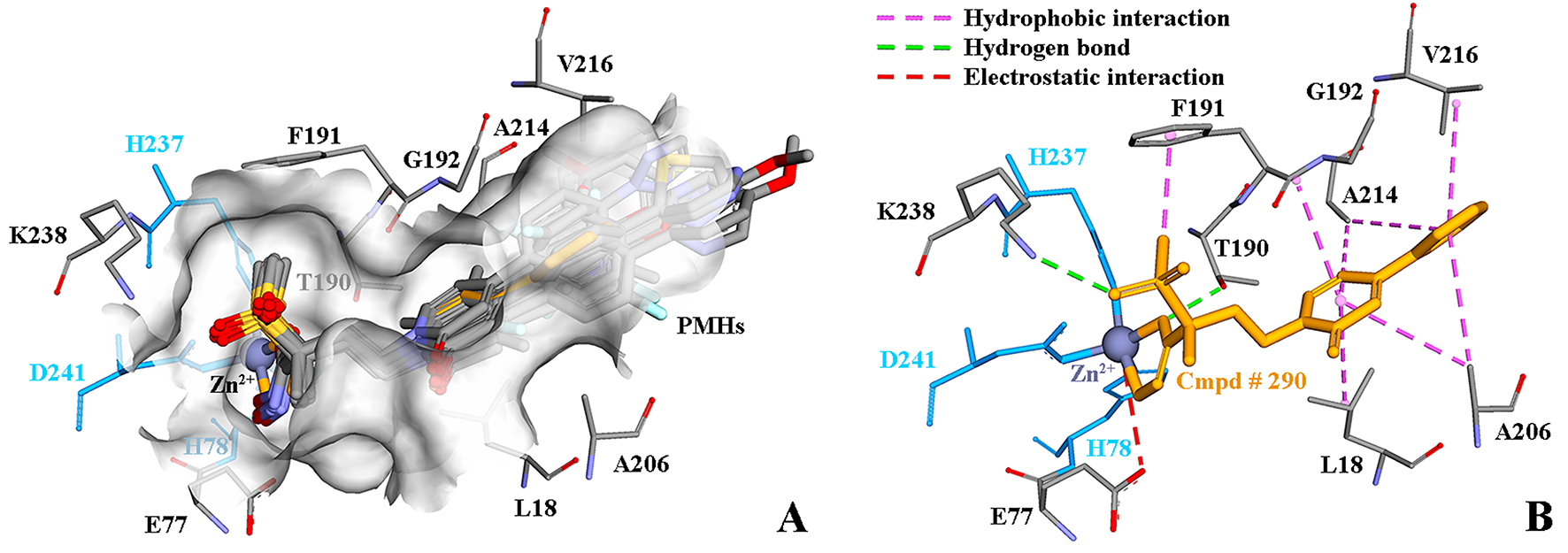


**Figure 5.** PMH LpxC inhibitors docking into the catalytic pocket (**A**); and the binding mode of the representative compound # 290 in the co-crystal structure (**B**).

2.6. Stability and Flexibility of Simulation Systems

Throughout the MD simulations, the potential energy of the PaLpxC and PaLpxC-Cmpd # 290 systems are nearly kept stable at −1.16 × 10^5^ kcal/mol, and the fluctuation rate is 0.172% (see Figure 6A). The root mean squared deviation (RMSD) analysis results of the systematic C_α_ atoms (see Figure 6B) shows that the RMSD of the PaLpxC system in the process of simulation is basically maintained at 1.124 ± 0.081 Å; whereas that of the complex system formed by PaLpxC and Cmpd # 290 is basically maintained at 1.135 ± 0.090 Å. For systems with more than 4764 atoms, low potential energy and RMSD values indicate that the MD simulation process is stable and reliable.

The root mean squared fluctuation (RMSF) of systematic C_α_ atoms is exhibited in Figure 6C. The RMSF value of C_α_ atoms indicates the flexibility of the corresponding amino acid residues. According to Figure 6C, there are three amino acid fragments of the PaLpxC molecule with RMSF values less than 0.5 nm, that is, H78–L86, I239–L248, and G263–A265. These three fragments are located near the catalytic Zn^2+^, and the lower flexibility is beneficial to the specific recognition of the substrate. Generally, the formation of the complex did not affect the RMSF value of the PaLpxC molecules, which indicates that the binding of the PMH LpxC inhibitor Cmpd # 290 produced no significant change in the flexibility of the PaLpxC molecules, and it may be because of the inherent low flexibility of the PaLpxC molecule itself.

The B-factor can be converted to RMSF by Equation (1):

| $\text{B}\text{-}\text{factor}=\frac{8}{3}\pi^{2}\left( \text{RMSF} \right)^{2}$ | (1) |
| --- | --- |

According to the correlation analysis of the B-factor and RMSF in Figure 6D, with a significant correlation and a correlation coefficient of fitting curve *r*^2^ = 0.30, the reliability of the MD simulation results is proven again.


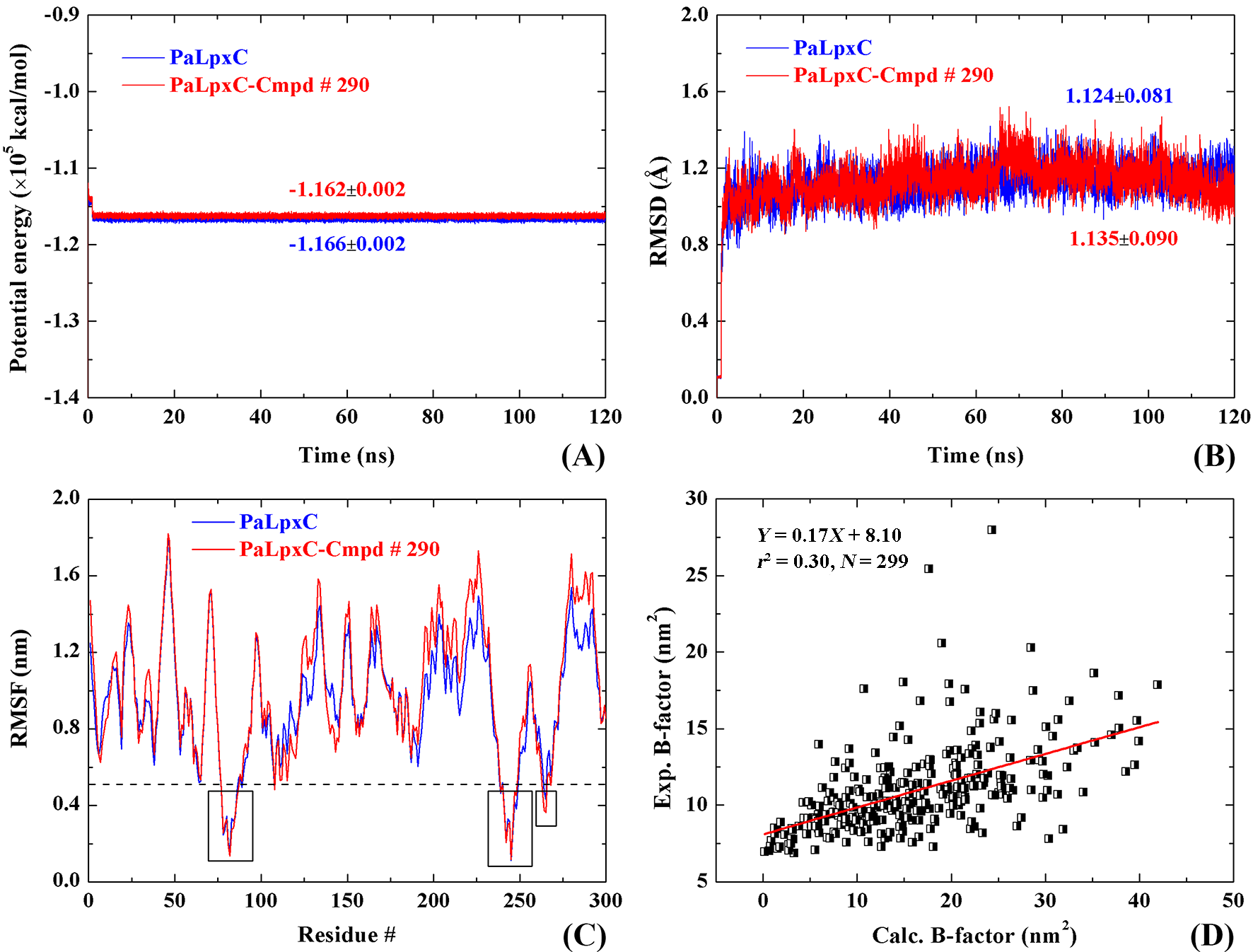


**Figure 6.** Potential energy (**A**); and root mean squared deviation (RMSD) (**B**) of the C_α_ atoms in the PaLpxC and PaLpxC-Cmpd # 290 versus simulation time, root mean squared fluctuation (RMSF) distribution (**C**) of the C_α_ atoms, and the correlation (**D**) of calculated B-factors with that from the PDB file 3UHM.pdb.

2.7. Molecular Recognition Mechanism

The H-bond is one of the most important non-bond interactions that maintains the stability of complex molecules, playing a key role in the process of molecular recognition. In order to further explore the molecular recognition mechanism between Cmpd # 290 and PaLpxC, Table S4 lists the H-bonds formed between Cmpd # 290 and PaLpxC. The H-bond is defined by geometry criterion [26]: the distance between the donor atom (D) and acceptor atom (A) is less than 3.5 Å, and the angle D-H-A is more than 135°.

As can be seen from Table S4, the stable H-bonds are formed between Cmpd # 290 and M62, T190, and H264 of PaLpxC. Miller's group [27] compared the co-crystal complex structure of *Aquifex* *aeolicus* LpxC (AaLpxC)-LPC-009 [28] with that of PaLpxC-LPC-009, proposing that the position of the carbonyl group in PaLpxC M62 is similar to that of AaLpxC H58, which is one of the key residues of the hydrolysis reaction. In the density functional theory (DFT) study [29] on the LpxC deacetylation mechanism, the formation of the H-bond between the hydroxyl at the side chain of T190 (residue number in PaLpxC) and the carbonyl oxygen atom of the substrate help to restrict it near the active site. In a series of studies of the AaLpxC hydrolysis mechanism [14,30,31], H264 (residue number in PaLpxC) is an important residue providing a proton for the amino group in hydrolysate. Thus, stable H-bonds between Cmpd # 290 and M62, T190, and H264 of PaLpxC play important roles in maintaining the stability of the complex, as well as the proton transfer process of the hydrolysis. It can also be seen from Table S4 that there is an H-bond formed between Cmpd # 290 and K238. Due to the rotation of the amino group, the H-bond between Cmpd # 290 and K238 is not as stable as the other H-bonds discussed above, with the total percentage of frames that showed the H-bond being only around 22.78%. Given that K238 is located in the uridine diphosphate (UDP) binding pocket [32,33], it is speculated that the H-bonds between Cmpd # 290 and K238 may be responsible for the high inhibitory capacity of Cmpd # 290.

Binding free energy is a dominant criterion in evaluating the activity of drug molecules. Molecular mechanics/Poisson-Boltzmann (MM/PBSA) algorithm was used to calculate the binding free energy. One snapshot was collected from the stable trajectory (20–100 ns) every 5 ns, and a total of 17 systemic conformations were collected from the MD trajectory for energy minimization. Each conformation was minimized to 100,000 steps with convergence of the energy gradient less than 0.0001 kcal·mol^−1^·Å^−2^ in entropy calculations. Table S5 lists the contributions to the binding free energy between PaLpxC and Cmpd # 290. In the form of each kind of energy item from Table S5, the free energy of PaLpxC decreased 13.79 kcal/mol after the formation of the complex with Cmpd # 290. The decrease of the systemic free energy is mainly due to two parts: the systemic van der Waals energy (*VDW*_IN_) and the hydrophobic part of the systemic solvation energy (*VDW*_PB_). From this point of view, the nonpolar interaction is the main driving force promoting the association of PaLpxC with the Cmpd # 290 inhibitor.

The energy decomposition results (see Figure 7 and Table S6) show that D241, F191, K238, M62, I197, T190, and H264 in the PaLpxC contribute greatly to the binding free energy. Combined with the analyses of the H-bond, it can be determined that M62, T190, K238, and H264 are the key residues in the molecular recognition between Cmpd # 290 and PaLpxC. According to the co-crystal complex structure of PaLpxC-Cmpd # 290 (see Figure 5B), D241 is also the key residue for fixing Zn^2+^ and maintaining its pentacoordinate configuration. Moreover, the phenyl of F191 also formed the π-alkyl mixed hydrophobic interaction with the methyl of Cmpd # 290. The detailed binding mode of Cmpd # 290 in the PaLpxC active pocket with the lowest energy conformation is shown in Figure 7.





**Figure 7.** Energy decomposition of the residues in PaLpxC.

2.8. Conformational Change of the Systems

FEL is a kind of probability distribution function of free energy obtained by principal component analysis and statistics based on MD equilibrium trajectories. Figure 8A,C shows the free energy distribution of PaLpxC-Cmpd # 290 and the PaLpxC systems at 300 K, respectively. The depth of the color in the figure represents the free energy level of the corresponding regions. According to Figure 8A, there are two lower free energy independent regions in the complex PaLpxC-Cmpd # 290, with M1 at the upper left and M2 at the upper right. The minimum free energy region mainly corresponds to 20–35 ns, 50–65 ns and 75–120 ns. There are also two independent lower free energy regions in the PaLpxC protein corresponding to M1 at the lower right and M2 at the upper left during 65–120 ns. Based on the distribution of the two reaction coordinates (i.e., PC1 and PC2), it is found that, after binding with the inhibitor Cmpd # 290, the low free energy region of the PaLpxC does not significantly increase or decrease, but the conformational sampling space reduces. Specifically, PC1 reduces from the original 0.70 to −0.70 to 0 to −0.60, indicating that the complex system is becoming more stable, which is consistent with the calculation results of free energy above (Figure S4).

In order to further analyze the local minimum area of the free energy distribution corresponding to simulation time, the conformational cluster analysis is performed with a cutoff of 1.1 Å for the PaLpxC-Cmpd # 290 and PaLpxC systems. The result is shown in Figure 8B,D. From Figure 8B,D, the number of clusters of the two systems is both two, and each cluster represents a relatively independent conformational mode. Statistical analysis shows that the distribution ratio of cluster 1 to cluster 2 of the PaLpxC protein is 25.2%:74.8%. After binding with Cmpd # 290, the corresponding distribution ratio changes to 21.1%:78.9%. The results indicate that the conformational transition space decreases in the complex system, and the association with Cmpd # 290 makes the system more stable. Figure S5 shows the slow-motion modes of PaLpxC (A) and PaLpxC-Cmpd # 290 (B). It can be seen from Figure S5 that after binding with the inhibitor Cmpd # 290, the local movement of the PaLpxC protein produce obvious changes. For example, the upper end of the α helices located in R1 (G263–A276) has a tendency to approach the inhibitor directly, as well as the β strand in the R2 (V137–A144) region. In addition, the rotational motion of the whole protein becomes slightly irregular and divergent after the association with inhibitors. It is speculated that the partial loss of the whole rotational motion and the contraction of R1/R2 local conformation both confirm the results of the PCA analyses. Interestingly, the key residues G263 and H264 obtained from energy decomposition calculation (see Figure 7 and Table S6) are located in the R1 region; nevertheless, the relationship between the motion mode change of PaLpxC and its binding with inhibitors remains to be further explored.


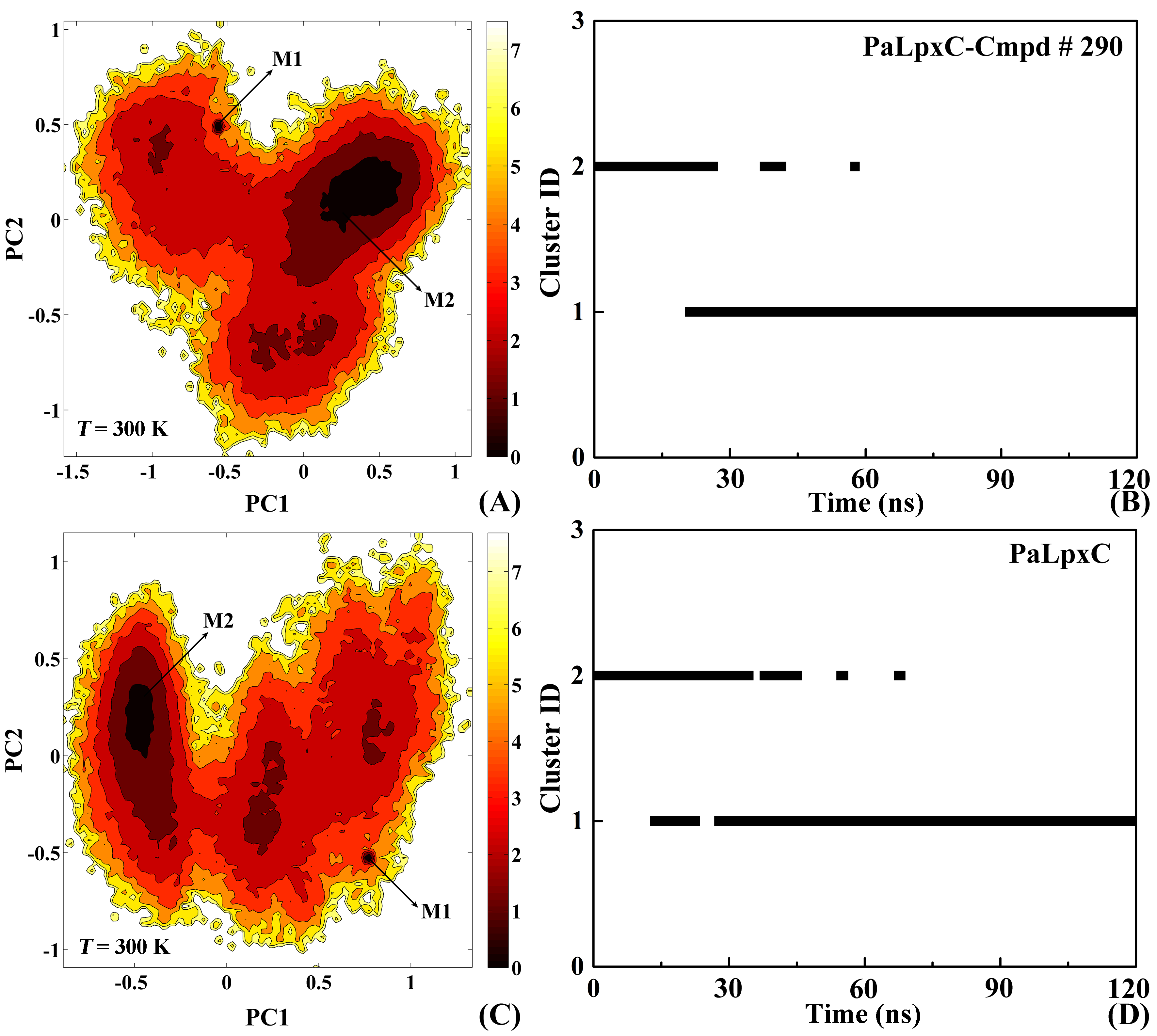


**Figure 8.** Free energy landscapes and the corresponding conformational cluster analyses for the PaLpxC-Cmpd # 290 (**A**,**B**); and PaLpxC protein (**C**,**D**) systems. M1 and M2 represent two independent minimal free energy regions, respectively. PC1: principal component 1; PC2: principal component 2.

2.9. Water Mediated H-Bonds

Water is an important reactant of the hydrolysis reaction catalyzed by PaLpxC. In order to further explore the possible inhibition mechanism of Cmpd # 290, the water molecules within 4 Å of the center of the active site of Zn^2+^ were sampled and analyzed before and after the association of PaLpxC with Cmpd # 290. The sampling interval is 400 ps and the sample capacity is about 300. Figure 9A shows the changes in the number of water molecules near the active site of PaLpxC in the absence of inhibitors. The result indicates that in the process of simulation, the number of water molecules within 4 Å around Zn^2+^ numbered between one and five. The probability of occurrence of one, two, three, four, and five water molecules are about 6.98%, 41.20%, 36.88%, 13.62%, and 1.33%, respectively. The same analysis of the PaLpxC-Cmpd # 290 complex system shows that with the presence of the inhibitor molecule, in the same range of the active site, there is no water molecule. This indicates that Cmpd # 290 can effectively prevent the water molecules from approaching the reaction center.

With the simulation trajectory of the PaLpxC system monitored by Visual Molecular Dynamics (VMD) package (University of Illinois at Urbana-Champaign, Urbana-Champaign, IL, USA), it is found that the variation of water molecules near the active site is obvious. Water molecules are present about 2.27 Å away from Zn^2+^ on average and tetracoordinate to Zn^2+^. It is consistent with the extended X-ray absorption fine structure (EXAFS) experimental results reported by McClure et al. [34]. In order to further discuss the binding characteristics of the water molecules in this area, we focused on the binding mode of the water molecules shown in Figure 9B. According to Figure 9B, in addition to coordinating with Zn^2+^, the water molecule also forms a stable H-bond with the carboxyl oxygen anion of E77, and a weak H-bond with H264. Previous studies [30,31,35] show that E77 serves as the key residue for the attack and deprotonation of the water molecule, leading to the hydrolysis reaction. By calculation, the distance between the carboxyl oxygen anion of E77 and the hydrogen atom of water molecules is about 1.73 Å, which is beneficial for the hydroxyl ion’s attack on the proton. The H-bond between H264 and the water molecule is another important factor stabilizing the hydroxyl ions, and assisting the nucleophilic attack on the acetyl of the natural substrate [35,36]. In addition, it is found that there is a certain interaction between the water molecule and M62 of the PaLpxC system. In some cases, an H-bond was also formed between the water molecule and T190. In general, the stability of the water molecule in this region mainly depends on the localization of multiple H-bonds near the active site of the PaLpxC system. The stable existence of these H-bonds is related to the inherent low flexibility of the amino acids, which is mentioned in the RMSF analysis above.


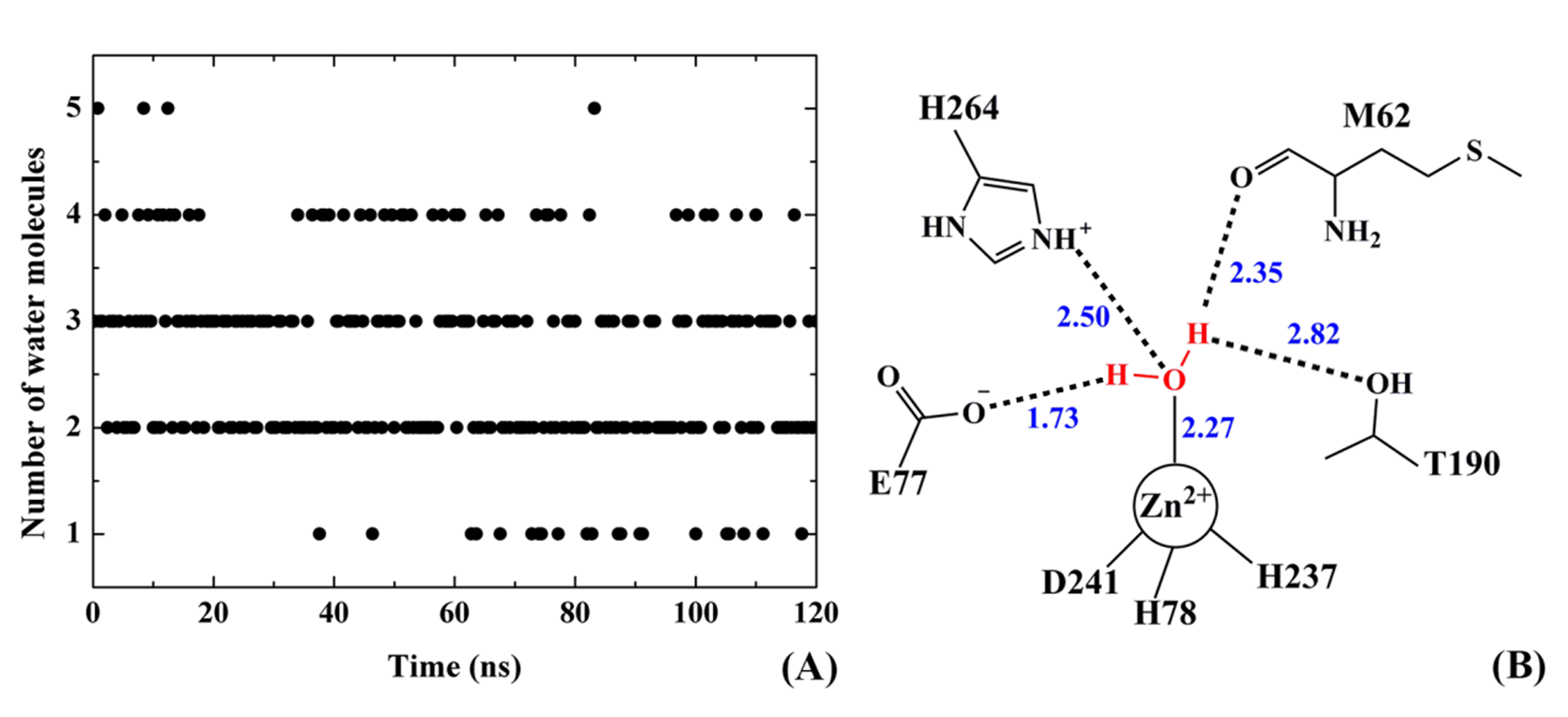


**Figure 9.** Variation of the number of water molecules within a 4 Å range near the active zinc ion of the PaLpxC system versus simulation time (**A**); and the binding mode of the key water molecule (**B**). The unit of distance between the two atoms exhibited in Figure B is Angstrom.

2.10. Possible Inhibitory Mechanism of Cmpd # 290

Based on the analyses of the molecular recognition between PaLpxC and Cmpd # 290, as well as the conformational change of the complex and the water molecules near the active site of PaLpxC, the following possible inhibition mechanism of Cmpd # 290 was proposed. Firstly, Cmpd # 290 is driven by nonpolar interactions, depending on the H-bond interaction with M62, T190, K238, and H264 in the active pocket, and influences the localization of water molecules at the catalytic center. After that, Cmpd # 290 chelates to Zn^2+^ with its hydroxamate moiety and forms a pentacoordination configuration, which prevents the water molecule from combining with it. Meanwhile, the hydrophobic terminus of Cmpd # 290 occupies the hydrophobic channel of PaLpxC via mixed hydrophobic interaction with F191 and I197, blocking the entry of other water molecules and natural substrates. Finally, the association with Cmpd # 290 also reduces the conformational transition space of PaLpxC, which makes the PaLpxC molecule more stable, ultimately resulting in inhibition (see Figure 10). In Figure 10, the crystal structure of the AaLpxC-substrate (green) was superimposed well onto the structure of the complex PaLpxC-Cmpd # 290 (cyan), due to high conservation. In the binding pocket, there is a large volume clash caused by Cmpd # 290 (blue) and the natural substrate (red) and the hydroxamate moiety chelates to Zn^2+^, thus occupying the coordination position of the water molecule (brown), which also leads to a clash at the hydrophilic terminus of Cmpd # 290.


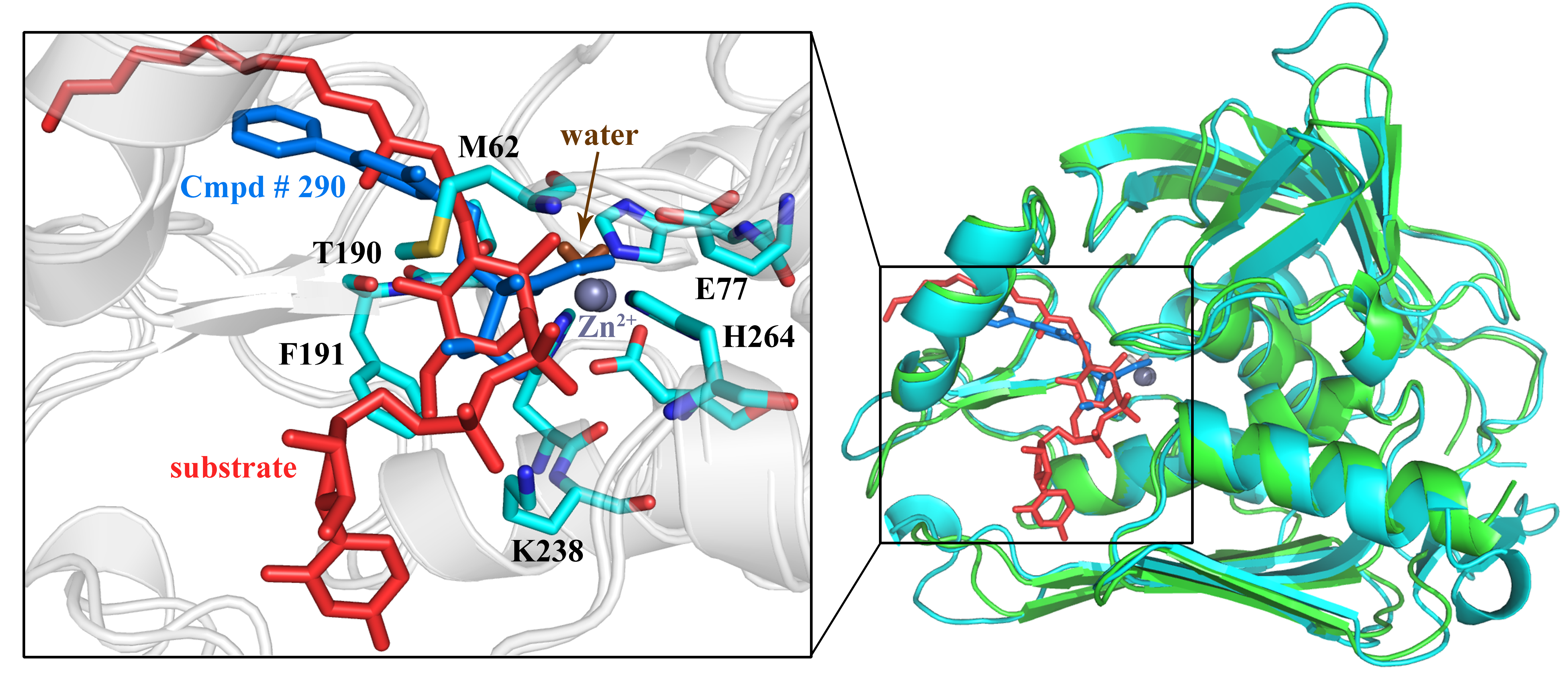


**Figure 10.** Superimposition of the crystal structure of the PaLpxC-Cmpd # 290 complex (cyan, PDB file: 3UHM.pdb) onto that of the AaLpxC-substrate complex (green, PDB file: 4OZE.pdb).

In summary, the possible structure-activity relationships of pyridone methylsulfone hydroxamate PaLpxC were proposed through 3D-QSAR modeling. Then, the binding mode of the complex PaLpxC-Cmpd # 290 and potential inhibitory mechanism of inhibitors both were explored theoretically. This work will provide a theoretical basis for the molecular design of LpxC-targeting antibiotics. Nevertheless, the essential biological experiment is decisive. We plan to gradually introduce relevant experiments in the follow-up work to verify these modeling results.

3. Materials and Methods

3.1. Molecular Docking

ChemBio3D Ultra 12.0 (Cambridge Soft, Cambridge, MA, USA) was used to construct the molecular structures of the PMH LpxC inhibitors except for Cmpd # 290, and energy optimization of these structures was performed under the MM2 molecular force field with the convergence condition of root mean square (RMS) less than 0.0001 kcal·mol^−1^·Å^−1^. After optimization, the structures were imported into SYBYL-X 1.3 [37] and molecular charges were calculated by the Gasteiger-Hückel method, and then another optimization under the Tripos force field was performed with the energy convergence condition of RMS less than 0.05 kcal·mol^−1^·Å^−1^ to generate the conformers for molecular docking. The AutoDock4.2 software package [38] was used to perform molecular docking, with sampling based on the Lamarckian genetic algorithm (LGA), total and local search results of energy combined, and the semi-empirical function of the binding free energy used to rank the intermolecular energy scores between the receptor and ligand. The docking force field used in this work was an exclusive force field developed for zinc [Zn(II) or Zn^2+^] containing systems (i.e., AutoDock4_Zn_ force field). In this force field, a pseudo-atom was introduced to guide the ligand coordinating with zinc, and the geometrical characteristics and bond strength of Zn(II) or Zn^2+^ were considered sufficiently, which greatly improved the accuracy of molecular docking for zinc-containing molecular systems [39]. In the current work, we determined the box center according to the Zn^2+^ coordinates of PaLpxC (PDB entry: 3UHM), and built a 40 Å × 90 Å × 60 Å rectangular box with a grid space of 0.375 Å. 128 conformations were collected in each docking, and the conformation of the lowest energy in the largest conformation cluster was defined as the near native conformation for the subsequent 3D-QSAR analyses.

3.2. 3D-QSAR

The PMH compounds were randomly divided into a training set (31 molecules in total) for constructing the 3D-QSAR models and a test set (7 molecules in total) for testing the predictive ability of the biological activity of the models. The IC_50_ value of the experiment was converted to a negative logarithm pIC_50_ form. Before constructing the 3D-QSAR models, it was necessary to align the compound molecules. The quality of molecular alignment has a great influence on the reliability of the 3D-QSAR models. In molecular alignment, ensuring all molecules using the construction of 3D-QSAR models with the largest similar spatial orientation of molecular fields was helpful to improve the similarity of the steric and electrostatic fields computed. Considering that the 3D-QSAR models assume that all molecules are in the same active binding mode at the same site, the crystallographic structure of Cmpd # 290 was selected as the template molecule, and molecular alignment was based on the pyridone methylsulfone hydroxamate commom substructure. 3D-QSAR analyses were completed in SYBYL-X 1.3 [37]. To set up the CoMFA model, the PMH molecules with alignment were placed in a spatial grid, a sp^3^ hybridized C^+^ probe walks in the space around the molecules, the interaction between the probe and molecules was calculated, and the energy values of the interaction with different space coordinates were recorded to obtain the steric and electrostatic field data; CoMSIA was similar to CoMFA, but in addition to the analyses of the steric field (S) and electrostatic field (E), the hydrophobic field (H), H-bond acceptor (A) field, and H-bond donor (D) field were also calculated.

The partial least squares (PLS) method was used to analyze the PMH molecules in training set. Firstly, the leave-one-out (LOO) method was used for cross validation (parameters are default) to obtain the optimal numbers of component (ONC) and the determination coefficient *q*^2^. Secondly, according to the ONC, the 3D-QSAR models were established by non-cross validation, and the correlation coefficient *r*^2^, standard error for estimate *E*_s_, root mean squared error (RMSE) and the *F*-test values *F* were obtained. Parameters based on the PLS analyses above can be used to evaluate the predictive ability and stability of the models, and predict the biological activity of the molecules in the test set. The predicted correlation coefficient for the test set *r*2 p was calculated by Equation (2):

| $r_{\text{p}}^{2}=\frac{\text{SD}-\text{PRESS}}{\text{SD}}$ | (2) |
| --- | --- |

In Equation (2), SD represents the sum of the squared deviations of the experimental biological activity of the test set and the average molecular biological activity of the training set, and PRESS indicates the sum of the squared errors of the predictive biological activity values of the test set and their biological activity as determined by the experiment.

3.3. MESP and Mulliken Charge Analyses

Gaussian 09 (Gaussian Inc., Wallingford, CT, USA) was used to calculate the MESP and Mulliken charges based on DFT. The molecular conformation of the ligand used in the calculation was generated by energy optimization, and the method and criterion of energy optimization were consistent with those of the previous molecular docking. The B3LYP/6-31G (d,p) basis set [40] was applied to optimize the geometry of the PMH LpxC inhibitors. In order to simulate the physiological environment as much as possible, all the energy was calculated in the solvent environment in the CPCM model [41]. The calculation of the MESP and Mulliken atomic charges was completed at the same theoretical level. The MESP iso-energy surface was superimposed by the color coded iso-potential surface onto the iso-electron density surface (0.001 e au^−3^). Different colors are used to represent the distribution of the MESP on the surface of the molecule. For example, dark red represents the region of the most negative electrostatic potential and dark blue represents the region of the most positive electrostatic potential.

3.4. Molecular Dynamics Simulation

The structural models of PaLpxC and complex PaLpxC-Cmpd # 290 were further subjected to MD simulations in the solution system for 120 ns, respectively, using the AMBER16 software package [42] with the AMBER ff14SB force field. The force field parameters were fitted based on the experimental results [43], and the simulated temperature was 300 K. The solvent was added with the TIP3P water model [44], and formed a 10 Å thick layer of water molecules on the surface of the systems. Using MCPB.py [45] to call Gaussian 09, according to the B3LYP method, the Zn^2+^ bond model and the force field parameters were constructed by using 6-31G* basis set. Energy minimization before simulation was completed by two steps. Firstly, with the constraint (constraint force is 500 kcal·mol^−1^·Å^−2^) of the solute, 5000 steps of the steepest descent method was performed and followed by another 5000 steps of the conjugate gradient method; then unrestrained minimizations of the whole system were executed by the same stages above with the convergence condition that the energy gradient is less than 0.01 kcal·mol^−1^·Å^−2^.

MD simulation consists of two steps: constrained MD simulation was performed for 1 ns with the constraint force constant at 10 kcal·mol^−1^·Å^−2^ and the temperature of the system gradually increased from 0 K to 300 K. Then free isothermal MD simulation of the systems was performed for 119 ns. The conformational changes in the whole process of simulations were tracked and observed by VMD, and using the SHAKE algorithm [46] to restrain the bond length, the radius of non-bond was 0.1 Å, and the time integration step was set to 2 fs. 12,768 and 11,162 water molecules were added to the PaLpxC system and the complex PaLpxC-Cmpd # 290 system. To balance the total charge of the systems, 7 and 6 Na^+^ were also added, and the total numbers of atoms in the PaLpxC and PaLpxC-Cmpd # 290 systems were 38,310 and 38,211, respectively.

3.5. Energy Decomposition Analysis

Energy decomposition analysis was performed by the molecular mechanics/generalized Born surface area (MM/GBSA) method [47]. The basic idea of this method is that: (1) the energy contribution of each residue can be divided into: the internal energy in a vacuum; the polar solvation energy calculated by the Generalized Born model [48]; and the nonpolar solvation energy calculated by LCPO algorithm [49]; (2) energy is decomposed onto the atoms of the backbone and side chain of each residue. Here, the internal energy in a vacuum can also be divided into two parts: polar electrostatic interaction and nonpolar van der Waals interaction. According to the LCPO algorithm, there is a positive correlation between nonpolar solvation energy and solvent accessible surface area (SASA). Thus, the contribution of the main residues in PaLpxC to the binding of Cmpd # 290 can be observed by MM/GBSA energy decomposition.

3.6. Free Energy Landscape Analysis

FEL [50] can promote our understanding of molecular recognition, folding, and aggregation by studying the free-energy minima and the free-energy barriers. The free-energy minima represent the conformational ensemble in stable states which are accessible to a biomolecule under physiological conditions and the free-energy barriers denote the transient states connecting them. The FEL can be constructed based on PCA [51]. The corresponding expression is

| $\Delta G\left( X \right)=-k_{\text{B}}TlnP\left( X \right)$ | (3) |
| --- | --- |

where *X* stands for the PCs and thus *P*(*X*) is the probability distribution of the molecular system along the PCs, and *k*_B_ and *T* are the Boltzmann constant and absolute temperature, respectively.

3.7. Conformational Cluster Analysis

On the basis of the conformations obtained by MD simulation, conformational cluster analysis was performed using MMTSB tools [52]. The specific analysis process is done according to the view of Daura et al. [53]: calculating the RMSD value between the conformations one by one, as a basis for the establishment of RMSD matrix (N × N, N stands for the numbers of conformations). After setting a RMSD threshold manually, and comparing any two RMSD values of conformations, if the difference is less than the threshold value, it is classified as a cluster. The lowest energy conformation in each cluster is used as the representative conformation. Here, the matrix was built by the RMSD of the whole system, and the situation of conformational cluster of overall systematic conformations is discussed.

4. Conclusions

In the current work, theoretical molecular simulation studies on a broad-spectrum LpxC inhibitor, a type of pyridone methylsulfone hydroxamate compound, were performed to investigate the relationship between its chemical structure and bioactivity. CoMFA and CoMSIA were used to study the 3D-QSAR of PMH LpxC, and QSAR models with good predictive ability were obtained. According to the results of the contour map, the introduction of bulky groups and electron withdrawing groups in PMH LpxC inhibitors can effectively improve the inhibitory activity of the inhibitors. Based on the analyses of the molecular electrostatic potential of the PMH LpxC inhibitors, the effect of substituents on the inhibitory activity was explained at the molecular level.

Comparative MD simulations of the PaLpxC and complex PaLpxC-Cmpd # 290 were carried out for 120 ns. The results of binding free energy calculations based on MD trajectories show that the binding free energy calculation results agree well with the experimental values, and the van der Waals interaction is the main driving force for the formation of the complex. The results of H-bond and energy decomposition show that Cmpd # 290 mainly interacts with M62, T190, F191, H264, I197, and K238 of PaLpxC.

Finally, through the simulation of trajectory sampling analyses, the binding mode of key water molecules in PaLpxC was investigated. Without any constraint, the water molecules can freely access the active pocket of PaLpxC; and with the assistance of E77, H264, T190, and M62, it can coordinate and form a tetrahedral configuration with Zn^2+^ (the average length of O-Zn is around 2.27 Å), to provide the reagent for the hydrolysis reaction constantly. In the complex structure, Cmpd # 290 chelates Zn^2+^ by hydroxamate moiety, which causes the destruction of the binding mode of water molecules. It further blocks the entry of the reaction substrate by enclosing the pocket through its hydrophobic chain, so as to exert an inhibitory effect. This work provides some theoretical guidance for the design of drug molecules targeting PaLpxC.

**Supplementary Materials:** Supplementary materials can be found at www.mdpi.com/link.

**Acknowledgments:** This work was supported by the funding from the National Natural Science Foundation of China (31600591, 11247018), the Science and Technology Bureau Foundation of Sichuan Province (2015JY0117), the Key Project of Sichuan Provincial Education Bureau (17ZA0194), the Project of Leshan Science and Technology Administration (14GZD022), The Project of Leshan Normal University (Z16019), The Projector of Sichuan Provincial Key Laboratory of natural product chemistry and small molecule catalysis (TRCWYXFZCH2016015).

**Author Contributions:** Conceived and designed the experiments: Ke Zuo, Xiaojun Gou, and Jianping Hu. Performed the experiments: Ke Zuo, Li Liang, Wenyi Du, and Jianping Hu. Analyzed the data: Ke Zuo, Li Liang, Wenyi Du, Xin Sun, and Wei Liu. Contributed analysis tools: Hua Wan and Jianping Hu. Wrote the paper:
Ke Zuo, Li Liang, Hua Wan, and Jianping Hu.

**Conflicts of Interest:** The authors declare no conflict of interest.

References

1. Paramythiotou, E.; Routsi, C. Association between infections caused by multidrug-resistant gram-negative bacteria and mortality in critically ill patients. *World* *J*. *Crit*. *Care* *Med*. **2016**, *5*, 111–120.
2. Basak, S.; Singh, P.; Rajurkar, M. Multidrug resistant and extensively drug resistant bacteria: A study. *J*. *Pathog*. **2016**, *2016*, 1–5.
3. Protic, D.; Pejovic, A.; Andjelkovic, D.; Djukanovic, N.; Savic, D.; Piperac, P.; Markovic, Denic, L.; Zdravkovic, M.; Todorovic, Z. Nosocomial infections caused by *Acinetobacter* *baumannii*: Are we losing the battle?. *Surg*. *Infect*. **2016**, *17*, 1–7.
4. Stoesser, N.; Mathers, A.J.; Moore, C.E.; Day, N.P.; Crook, D.W. Colistin resistance gene *MCR-1* and pHNSHP45 plasmid in human isolates of *Escherichia* *coli* and *Klebsiella* *pneumoniae*. *Lancet* *Infect*. *Dis*. **2016**, *16*, 285–286.
5. Labarca, J.A.; Salles, M.J.C.; Seas, C.; Guzmán-Blanco, M. Carbapenem resistance in *Pseudomonas* *aeruginosa* and *Acinetobacter* *baumannii* in the nosocomial setting in Latin America. Critical *Rev*. *Microbiol*. **2016**, *2*, 276–292.
6. Konya, V.; Maric, J.; Jandl, K.; Luschnig, P.; Aringer, I.; Lanz, I.; Platzer, W.; Theiler, A.; Bärnthaler, T.;
   Frei, R.; et al. EP4 receptor prevents endotoxin-induced neutrophil infiltration into the airways and enhances microvascular barrier function. *Br*. *J*. *Pharmacol*. **2015**, *172*, 4454–4468.
7. Murzyn, K.; Pasenkiewiczgierula, M. Structural properties of the water/membrane interface of a bilayer built of the *E*. *coli* Lipid A. *J*. *Phys*. *Chem*. *B* **2015**, *119*, 5846–5856.
8. Anderson, M.S.; Bull, H.G.; Galloway, S.M.; Kelly, T.M.; Mohan, S.; Radika, K.; Raetz, C.R. UDP-*N*-acetylglucosamine acyltransferase of *Escherichia* *coli*. The first step of endotoxin biosynthesis is thermodynamically unfavorable. *J*. *Biol*. *Chem*. **1993**, *268*, 19858–19865.
9. Emiola, A.; George, J.; Andrews, S.S. A complete pathway model for lipid A biosynthesis in *Escherichia* *coli*. *PLoS* *ONE* **2015**, *10*, e0121216.
10. Mcclerren, A.L.; Stephanie, E.; Bowman, J.L.; Andersen, N.H.; Guan, Z.; Rudolph, J.; Raetz, C.R. A slow, tight-binding inhibitor of the zinc-dependent deacetylase LpxC of lipid A biosynthesis with antibiotic activity comparable to ciprofloxacin. *Biochemistry* **2006**, *44*, 16574–16583.
11. RCSB. Available online: www.rcsb.org (accessed on 30 February 2017).
12. Jackman, J.E.; Raetz, C.R.; Fierke, C.A. Site-directed mutagenesis of the bacterial metalloamidase UDP-(3-*O*-Acyl)-*N*-acetylglucosamine deacetylase (LpxC). Identification of the zinc binding site. *Biochemistry* **2001**, *40*, 514–523.
13. Whittington, D.A.; Rusche, K.M.; Shin, H.; Fierke, C.A.; Christianson, D.W. Crystal structure of LpxC, a zinc-dependent deacetylase essential for endotoxin biosynthesis. *Proc*. Natl. *Acad*. *Sci*. *USA* **2003**, *100*, 8146–8150.
14. Coggins, B.E.; Mcclerren, A.L.; Jiang, L.; Li, X.; Rudolph, J.; Hindsgaul, O.; Raetz, C.R.; Zhou, P. Refined solution structure of the LpxC-TU-514 complex and pKa analysis of an active site histidine: Insights into the mechanism and inhibitor design. *Biochemistry* **2005**, *44*, 1114–1126.
15. Coggins, B.E.; Li, X.; Mcclerren, A.L.; Hindsgaul, O.; Raetz, C.R.; Zhou, P. Structure of the LpxC deacetylase with a bound substrate-analog inhibitor. *Nat*. *Struct*. *Biol*. **2003**, *10*, 645–651.
16. Kalinin, D.V.; Holl, R. Insights into the zinc-dependent deacetylase LpxC: Biochemical properties and inhibitor design. *Curr*. *Top*. *Med*. *Chem*. **2016**, *16*, 2379–2430.
17. Tangherlini, G.; Torregrossa, T.; Agoglitta, O.; Köhler, J.; Melesina, J.; Sippl, W.; Holl, R. Synthesis and biological evaluation of enantiomerically pure glyceric acid derivatives as LpxC inhibitors. Bioorganic *Med*. *Chem*. **2016**, *24*, 1032–1044.
18. Müller, H.; Gabrielli, V.; Agoglitta, O.; Holl, R. Chiral pool synthesis and biological evaluation of C-furanosidic and acyclic LpxC inhibitors. *Eur*. *J*. *Med*. *Chem*. **2016**, *110*, 340–375.
19. Jana, S.K.; Löppenberg, M.; Daniliuc, C.G.; Holl, R. Synthesis and biological evaluation of C-ethynyl furanosides as LpxC inhibitors. *Tetrahedron* **2015**, *71*, 956–966.
20. Kurasaki, H.; Tsuda, K.; Shinoyama, M.; Takaya N.; Yamaguchi, Y.; Kishii, R.; Iwase, K.; Ando, N.; Nomura, M.; Kohno, Y. LpxC inhibitors: Design, synthesis, and biological evaluation of oxazolidinones as Gram-negative antibacterial agents. *ACS* *Med*. *Chem*. *Lett*. **2016**, *7*, 623–628.
21. Titecat, M.; Liang, X.; Lee, C.J.; Charlet, A.; Hocquet, D.; Lanbert, T.; Pagès, J.M.; Courcol, R.; Sebbane, F.; Toone, E.J.; et al. High susceptibility of MDR and XDR Gram-negative pathogens to biphenyl-diacetylene-based difluoromethyl-allo-threonyl-hydroxamate LpxC inhibitors. *J*. *Antimicrob.* *Chemother.* **2016**, *71*, 2874–2882.
22. Abdel-Magid, A.F. LpxC inhibitors as effective therapy against multidrug resistant bacterial infections. *ACS* *Med*. *Chem*. *Lett*. **2015**, *6*, 1095–1096.
23. Ding, S.; Wang, W.K.; Cao, Q.; Chu, W.J.; Lan, L.F.; Hu, W.H.; Yang, Y.S. Design, synthesis and biological evaluation of LpxC inhibitors with novel hydrophilic terminus. *Chin*. *Chem*. *Lett*. **2015**, *26*, 763–767.
24. Montgomery, J.I.; Brown, M.F.; Reilly, U.; Price, L.M.; Abramite, J.A.; Barham, R.; Che, Y.; Chen, J.M.; Chung, S.W.; Collantes, E.M.; et al. Pyridone methylsulfone hydroxamate LpxC inhibitors for the treatment of serious Gram-negative infections. *J*. *Med*. *Chem*. **2012**, *55*, 1662–1670.
25. ChEMBL. Available online: www.ebi.ac.uk/chembl (accessed on 30 February 2017).
26. Hu, J.P.; Gong, X.Q.; Su, J.G.; Chen, W.Z.; Wang, C.X. Study on the molecular mechanism of inhibiting HIV-1 integrase by EBR28 peptide via molecular modeling approach. *Biophys*. *Chem*. **2008**, *132*, 69–80.
27. Miller, M.D.; Ning, G.; Ross, P.L.; Olivier, N.B. Crystal structure of *A*. *aeolicus*, LpxC with bound product suggests alternate deacetylation mechanism. *Proteins* *Struct*. *Funct*. *Bioinform*. **2015**, *83*, 1706–1719.
28. Lee, C.L.; Liang, X.; Chen, X.; Zheng, D.; Sang, H.J.; Chung, H.S.; Barb, A.W.; Swanson, S.M.;
    Nicholas, R.A.; Li, Y.; et al. Species-specific and inhibitor-dependent conformations of LpxC: Implications for antibiotic design. *Chem*. *Biol*. **2011**, *18*, 38–47.
29. Robinet, J.J.; Gauld, J.W. DFT investigation on the mechanism of the deacetylation reaction catalyzed by LpxC. *J*. *Phys*. *Chem*. *B* **2008**, *112*, 3462–3469.
30. Hernick, M.; Gennadios, H.A.; Whittington, D.A.; Rusche, K.M.; Christianson, D.W.; Fierke, C.A. UDP-3-*O*-((*R*)-3-hydroxymyristoyl)-*N*-acetylglucosamine deacetylase functions through a general acidbase catalyst pair mechanism. *J*. *Biol*. *Chem*. **2005**, *280*, 16969–16978.
31. McClerren, A.L.; Zhou, P.; Guan, Z.; Raetz, C.R.; Rudolph, J. Kinetic analysis of the zinc-dependent deacetylase in the lipid A biosynthetic pathway. *Biochemistry* **2005**, *44*, 1106–1113.
32. Barb, A.W.; Zhou, P. Mechanism and inhibition of LpxC: An essential zinc-dependent deacetylase of bacterial lipid A synthesis. *Curr*. *Pharm*. *Biotechnol*. **2008**, *9*, 9–15.
33. Hale, M.R.; Hill, P.; Lahiri, S.; Miller, M.D.; Ross, P.; Alm, R.; Gao, N.; Kutschke, A.; Johnstone, M.; Prince, B.; Thresher, J.; Yang, W. Exploring the UDP pocket of LpxC through amino acid analogs. *Bioorganic Med*. *Chem*. *Lett*. **2013**, *23*, 157–158.
34. McClure, C.P.; Rusche, K.M.; Peariso, K.; Jackman, J.E.; Fierke, C.A.; Penner-Hahn, J.E. EXAFS studies of the zinc sites of UDP-(3-Oacyl)-*N*-acetylglucosamine deacetylase. *J*. *Inorg.* *Biochem*. **2003**, *94*, 78–85.
35. Clayton, G.M.; Klein, D.J.; Rickert, K.W.; Patel, S.B.; Kornienko, M.; Zugay-Murphy, J.; Reid, J.C.; Tummala, S.; Sharma, S.; Singh, S.B.; et al. Structure of the bacterial deacetylase LpxC bound to the nucleotide reaction product reveals mechanisms of oxyanion stabilization and proton transfer. *J*. *Biol*. *Chem*. **2013**, *288*, 34073–34080.
36. Gennadios, H.A.; Whittington, D.A.; Li, X.; Fierke, C.A.; Christianson, D.W. Mechanistic inferences from the binding of ligands to LpxC, a metal-dependent deacetylase. *Biochemistry* **2006**, *45*, 7940–7948.
37. *SYBYL-X*, version 1.3; Tripos International: St. Louis, MO, USA, 2010.
38. Hu, J.P.; He, H.Q.; Tang, D.Y.; Sun, G.F.; Zhang, Y.Q.; Fan, J.; Chang, S. Study on the interactions between diketo-acid inhibitors and prototype foamy virus integrase-DNA complex via molecular docking and comparative molecular dynamics simulation methods. *J*. *Biomol*. *Struct*. *Dyn*. **2013**, *31*, 734–747.
39. Santos-Martins, D.; Forli, S.; Ramos, M.J.; Olson, A.J. AutoDock4_Zn_: An improved AutoDock force field for small-molecule docking to zinc metalloproteins. *J*. *Chem*. *Inf*. *Model*. **2014**, *54*, 2371–2379.
40. Becke, A.D. Density-functional thermochemistry. III. The role of exact exchange. *J*. *Chem*. *Phys.* **1993**, *98*, 5648–5652.
41. Cossi, M.; Rega, N.; Scalmani, G.; Barone, V. Energies, structures, and electronic properties of molecules in solution with the C-PCM solvation model. *J*. *Comput*. *Chem*. **2003**, *24*, 669–681.
42. Case, D.A.; Betz, R.M.; Botello-Smith, W.; Cerutti, D.S.; Cheatham, III, T.E.; Darden, T.A.; Duke, R.E.; Giese, T.J.; Gohlke, H.; Goetz, A.W.; et al. *AMBER 2016*; University of California: San Francisco, CA, USA, 2016.
43. Wang, J.; Wolf R.M.; Caldwell, J.W.; Kollman, P.A.; Case, D.A. Development and testing of a general amber force field. *J*. *Comput*. *Chem*. **2004**, *25*, 1157–1174.
44. Jorgensen, W.L.; Chandrasekhar, J.; Madura, J.D.; Impey, R.W.; Klein, M.L. Comparison of simple potential functions for simulating liquid water. *J*. *Chem*. *Phys*. **1983**, *79*, 926–935.
45. Li, P.; Merz, K.M. MCPB.py: A python based metal center parameter builder. *J*. *Chem*. *Inf*. *Model*. **2016**, *56*, 599–604.
46. Ryckaert, J.P.; Ciccotti, G.; Berendsen, H.J. C. Numerical integration of the cartesian equations of motion of a system with constraints: Molecular dynamics of n-alkanes. *J*. *Comput*. *Phys*. **1977**, *23*, 327–341.
47. Kollman, P.A.; Massova, I.; Reyes, C.; Kuhn, B.; Huo, S.; Chong, L.; Lee, M.; Lee, T.; Duan, Y.;
    Wang, W.; et al. Calculating structures and free energies of complex molecules: Combining molecular mechanics and continuum models. *Acc*. *Chem*. *Res*. **2000**, *33*, 889–897.
48. Simonson, T. Macromolecular electrostatics: Continuum models and their growing pains. *Curr*. *Opin*. *Struct*. *Biol*. **2001**, *11*, 243–252.
49. Weiser, J.; Shenkin, P.S.; Still, W.C. Approximate atomic surfaces from linear combinations of pairwise overlaps (LCPO). *J*. *Comput*. *Chem*. **1999**, *20*, 217–230.
50. Nguyen, P.H.; Derrenumaux, P. Configurational entropy: An improvement of the quasiharmonic approximation using configurational temperature. *Phys*. *Chem*. *Chem*. *Phys*. **2012**, *14*, 877–886.
51. Barlow, P.N.; Steinkasserer, A.; Norman, D.G.; Kieffer, B.; Wiles, A.P.; Sim, R.B.; Campbell, I.D. Solution structure of a pair of complement modules by nuclear magnetic resonance. *J*. *Mol*. *Biol*. **1993**, *232*, 268–284.
52. Feig, M.; Karanicolas, J.; Brooks, C.L., III; MMTSB tool set: Enhanced sampling and multiscale modeling methods for applications in structural biology. *J*. *Mol*. *Graph*. Model. **2004**, *22*, 377–395.
53. Daura, X.; Gademann, K.; Jaun, B.; Seebach, D.; van Gunsteren, W.F.; Mark, A.E. Peptide folding: When simulation meets experiment. *Angew.* *Chem.* *Int.* *Ed*. **1999**, *38*, 236–240.

© 2017 by the authors. Submitted for possible open access publication under the
terms and conditions of the Creative Commons Attribution (CC BY) license (http://creativecommons.org/licenses/by/4.0/).
